# Uncertainty in the Bayesian meta-analysis of normally distributed surrogate endpoints

**DOI:** 10.1177/0962280215597260

**Published:** 2015-08-13

**Authors:** Sylwia Bujkiewicz, John R Thompson, Enti Spata, Keith R Abrams

**Affiliations:** 1 Biostatistics Research Group, Department of Health Sciences, University of Leicester, University Road, Leicester, UK; 2 Genetic Epidemiology Group, Department of Health Sciences, University of Leicester, University Road, Leicester, UK

**Keywords:** Meta-analysis, surrogate endpoints, Bayesian statistics, bivariate meta-analysis, meta-regression

## Abstract

We investigate the effect of the choice of parameterisation of meta-analytic models and related uncertainty on the validation of surrogate endpoints. Different meta-analytical approaches take into account different levels of uncertainty which may impact on the accuracy of the predictions of treatment effect on the target outcome from the treatment effect on a surrogate endpoint obtained from these models. A range of Bayesian as well as frequentist meta-analytical methods are implemented using illustrative examples in relapsing–remitting multiple sclerosis, where the treatment effect on disability worsening is the primary outcome of interest in healthcare evaluation, while the effect on relapse rate is considered as a potential surrogate to the effect on disability progression, and in gastric cancer, where the disease-free survival has been shown to be a good surrogate endpoint to the overall survival. Sensitivity analysis was carried out to assess the impact of distributional assumptions on the predictions. Also, sensitivity to modelling assumptions and performance of the models were investigated by simulation. Although different methods can predict mean true outcome almost equally well, inclusion of uncertainty around all relevant parameters of the model may lead to less certain and hence more conservative predictions. When investigating endpoints as candidate surrogate outcomes, a careful choice of the meta-analytical approach has to be made. Models underestimating the uncertainty of available evidence may lead to overoptimistic predictions which can then have an effect on decisions made based on such predictions.

## 1 Introduction

Biomarkers and surrogate endpoints are increasingly being investigated as candidate endpoints in clinical trials where measuring a primary outcome of interest may be too costly, too difficult or require a long follow-up time. Use of surrogate endpoints in clinical trial design has advantages in overcoming these difficulties by choosing more convenient, cheaper or shorter term endpoints. Such endpoints are also becoming increasingly important in health technology assessment (HTA) and in particular in the early stages of drug development when conditional licensing based on a biomarker takes place and evidence on treatment effectiveness on a target outcome may be limited. Suitable methods need to be identified that would incorporate data on surrogate outcomes most efficiently in evidence synthesis as part of HTA.

Validating candidate outcomes as surrogate endpoints to target outcomes requires the correlation between the candidate endpoint and the target outcome on the individual level as well as the correlation between the treatment effect measured by the surrogate endpoint and the treatment effect measured by the target outcome to be established.^1^ Methods for evaluating surrogacy on the individual level include, for example, Prentice's criteria,^2^ proportion of treatment explained^3^ and adjusted association (between the endpoints adjusted for the treatment).^4^ For the evaluation to be valid in a general context of a particular disease area, it needs to be performed on a number of studies rather than based on a single trial. Meta-analysis serves the purpose of combining evidence from a number of trials and also provides a convenient tool for evaluating the association between treatment effects on the surrogate and final outcome on the study level. A number of meta-analytical methods have been proposed that aim to validate such surrogate endpoints.^1,5,6^ For example, Daniels and Hughes proposed a Bayesian model for a joint synthesis of correlated outcomes, focused on summary data where partially available patient data can contribute to determining the within-study correlation.^6^ Buyse et al., on the other hand, designed a frequentist meta-analytic model based on patient-level data from a number of studies in the form of a mixed effects model with two measures of surrogacy derived: on the patient level and the study level.^5^ Part of the validation process, beyond establishing the correlations on both levels, involves investigating whether the treatment effect measured by the target outcome can be predicted from the treatment effect measured by the surrogate endpoint (from a model built based on treatment effect on both outcomes measured in historical trials) by comparing the predicted effect with the observed effect on a target endpoint in a validation study. Methods used for prediction include linear regression (for example proposed by Buyse et al. to predict the log hazard ratio measured by overall survival from the log hazard ratio measured by progression-free survival in colorectal cancer^7^), weighted linear regression (for example by Sormani et al.^8^ in a study in relapsing–remitting multiple sclerosis (RRMS)), error-in-variables regression methods^1^ (for example used by Burzykowski et al. in metastatic breast cancer study^9^ or Oba et al. in gastric cancer study^10^), meta-regression (for example used by Gabler et al. investigating 6 min walk distance as a surrogate endpoint to development of clinical events in pulmonary arterial hypertension^11^), or bivariate meta-analysis methods, such as by Daniels and Hughes in a Bayesian framework developed to evaluate CD4 cell count as a candidate surrogate endpoint for the treatment effect on the development of AIDS or death.^6^

Different meta-analytical approaches take into account different levels of uncertainty which may impact on the accuracy of the validation and predictions. The aim of this study was to investigate the effect of the choice of parameterisation of meta-analytic models and related uncertainty (that these models allow to incorporate) on the predictions obtained from those models. Bayesian methods are most suitable for this purpose as they are flexible in modelling the uncertainty. This study is concerned with predictive models for normally distributed treatment effects that are based on the summary data only. A range of Bayesian meta-analytical methods (using summary data) is implemented in order to investigate the impact of the choice of a model and level of uncertainty on the model predictions. When simple meta-regression is used to validate a candidate surrogate endpoint, the treatment effect on such an endpoint is included in the model as a covariate and hence is incorporated with no uncertainty, while the effect of treatment on each endpoint, including the surrogate, is in fact measured with error. Two approaches to meta-regression (described in Section 3.1) are investigated here: a standard use of mean trend with fixed coefficients estimated from the fixed effects meta-regression model (FEMR) and a random effects approach where between-study variability is taken into account when making predictions. In contrast to the meta-regression, the model proposed by Daniels and Hughes^6^ (described in Section 3.2) includes the treatment effect on the surrogate endpoint with uncertainty by modelling it as a response (rather than a covariate). Alternatively this can be achieved using bivariate meta-analytic methods^12–14^ (Sections 3.3 and 3.4) which allow one to simultaneously model the estimates of treatment effects on both the surrogate and the final endpoint by taking into account the between- and within-study correlations. Models are implemented using WinBUGS.^15^ While, as noted above, Bayesian methods are most suited to flexibly model the uncertainty, similar differences in the way uncertainty is taken into account and the impact of it on predictions can be also demonstrated using frequentist methods. We illustrate this by the use of meta-regression and bivariate meta-analysis in Stata.^16^

In the remainder of this paper, illustrative examples in RRMS and gastric cancer are introduced in Section 2, followed by the details of each model described in the Bayesian framework in Section 3, with additional details of the use of frequentist methods in Section 3.7 and methods for surrogate endpoint validation and model comparison in Section 3.8. Results are then presented and differences between the models discussed in Section 4 which are complemented by a simulation study in Section 5 aiming to test the performance of each method and its sensitivity to the distributional assumptions. The paper is concluded by a discussion section. WinBUGS coding for each of the models, R code for the simulation and Stata code for the frequentist approach are included in Appendix 1.

## 2 Illustrative examples

### 2.1 Multiple sclerosis

Sormani et al.^8^ showed that in studies investigating treatment effect in patients with multiple sclerosis, the treatment effect on relapse rate can potentially be used as a surrogate endpoint to the treatment effect on the disability progression rate. We use data from this study as an illustrative example to investigate the effect of the choice of modelling technique and corresponding level of uncertainty which is allowed to be included in each of the models. We refer to these data as the ‘Sormani data’ in the remainder of this paper.

**Figure 1.**
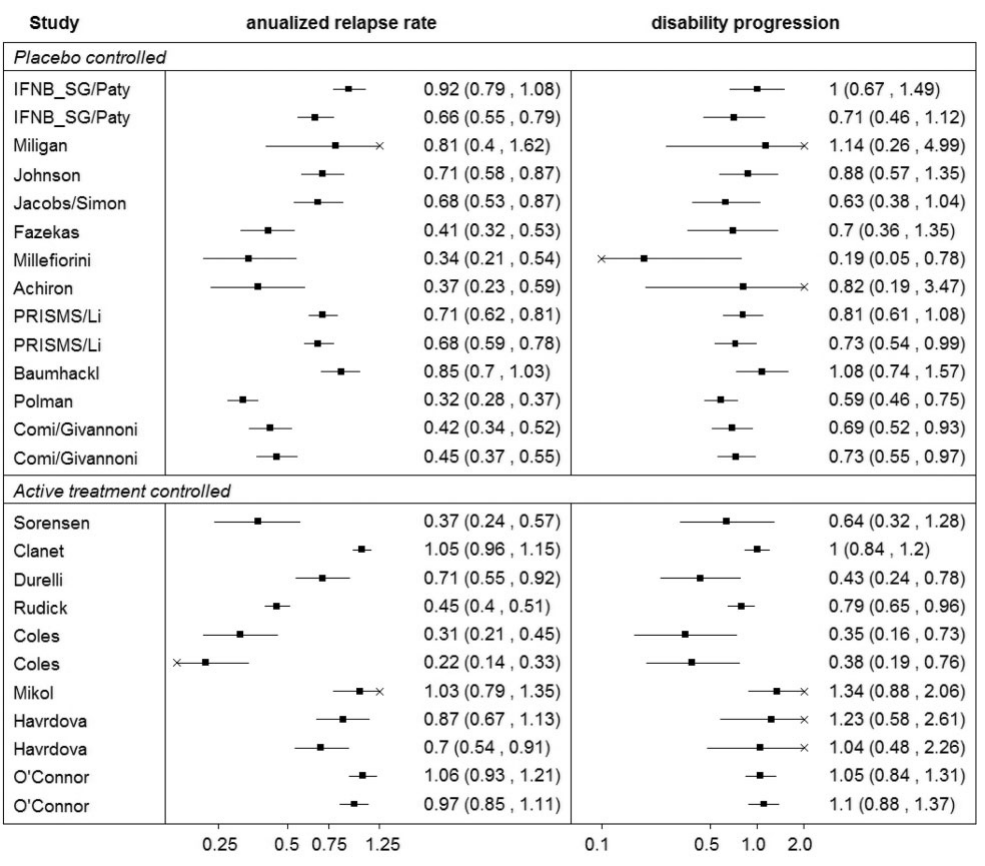
Summary of the ‘Sormani data’.

The annualised relapse rate ratio, the ratio between the relapse rate in the experimental and the control arms, was used as the summary estimate of the treatment effect on relapses (the surrogate endpoint measuring the treatment effect). The disability progression rate ratio, the ratio between the proportion of patients with a disability progression in the experimental and the control arms at year 2 (or at year 3 for trials of longer follow-up time which do not report the outcome at year 2), was used as the summary estimate of the treatment effect on disability progression, which was the target endpoint. Details of the specific treatment regimens are included in Table 1. Figure 1 shows data on both outcomes graphically, revealing similar heterogeneity patterns between the studies for both outcomes, implying a strong correlation between the effects on these outcomes. The studies are grouped as placebo-controlled and active-treatment-controlled.

**Table 1.** Studies in the ‘Sormani data’ reporting the annualised relapse rate ratio and the disability progression rate ratio.

Study	Contrast	Number	Follow-up	Annualised relapse	Disability progression
		of patients	(months)	rate ratio	rate ratio
Paty (1) 1993	IFNbeta-1b 1.6 MIU vs PBO	248	24	0.92 (0.82, 1.03)	1.00 (0.67, 1.49)
Paty (2) 1993	IFNbeta-1b 8 MIU vs PBO	247	24	0.66 (0.58, 0.75)	0.71 (0.46, 1.12)
Miligan 1994	Methylprednisolone vs PBO	26	24	0.81 (0.50, 1.30)	1.14 (0.26, 5.03)
Johnson 1995	GA vs PBO	251	24	0.71 (0.61, 0.82)	0.88 (0.57, 1.35)
Jacobs 1996	IFNbeta-1a 6 MIU vs PBO	172	24	0.68 (0.57, 0.81)	0.63 (0.38, 1.04)
Fazekas 1997	IVIg vs PBO	150	24	0.41 (0.34, 0.49)	0.70 (0.36, 1.35)
Millefiorini 1997	Mitoxantrone vs PBO	51	24	0.34 (0.24, 0.47)	0.19 (0.05, 0.78)
Achiron 1998	IVIg vs PBO	40	24	0.37 (0.27, 0.52)	0.82 (0.19, 3.50)
Li (1) 1998	IFNbeta1a 22 *μ*g vs PBO	376	24	0.71 (0.64, 0.78)	0.81 (0.61, 1.08)
Li (2) 1998	IFNbeta1a 44 *μ*g vs PBO	371	24	0.68 (0.62, 0.75)	0.73 (0.54, 0.99)
Baumhackl 2005	Hydrolytic enzymes vs PBO	306	24	0.85 (0.74, 0.97)	1.08 (0.74, 1.57)
Polman 2006	NAT vs PBO	942	24	0.32 (0.29, 0.36)	0.59 (0.46, 0.75)
Comi (1) 2009	Cladribine 3.5 mg/kg vs PBO	870	24	0.42 (0.36, 0.49)	0.69 (0.52, 0.93)
Comi (2) 2009	Cladribine 5.25 mg/kg vs PBO	893	24	0.45 (0.39, 0.52)	0.73 (0.55, 0.97)
Sorensen 2009	IFNbeta-1a and oral methylprednisolone	130	24	0.37 (0.27, 0.50)	0.64 (0.32, 1.28)
	vs IFNbeta-1a and PBO				
Clanet 2002	IFNbeta-1a 60 *μ*g vs 30*μ*g	802	36	1.05 (0.99, 1.12)	1.00 (0.84, 1.20)
Durelli 2002	IFNbeta1b vs IFNbeta1a	188	24	0.71 (0.59, 0.86)	0.43 (0.24, 0.78)
Rudick 2006	NAT + IFNbeta-1a vs IFNbeta-1a	1171	24	0.45 (0.41, 0.49)	0.79 (0.65, 0.96)
Coles (1) 2008	ALE 12 mg vs IFNbeta-1a	223	36	0.31 (0.24, 0.40)	0.35 (0.16, 0.73)
Coles (2) 2008	ALE 24 mg vs IFNbeta-1a	221	36	0.22 (0.16, 0.30)	0.38 (0.19, 0.76)
Mikol 2008	IFNbeta vs GA	764	24	1.03 (0.90, 1.17)	1.34 (0.88, 2.06)
Havrdova (1) 2009	IFNbeta-1a 30 *μ*g plus AZA 50 mg	118	24	0.87 (0.73, 1.04)	1.23 (0.58, 2.62)
	vs IFNbeta-1a 30 *μ*g				
Havrdova (2) 2009	IFNbeta-1a 30 *μ*g IM plus AZA 50 mg plus	123	24	0.70 (0.58, 0.85)	1.04 (0.48, 2.27)
	prednisone 10 mg vs IFNbeta-1a 30 *μ*g				
O'Connor (1) 2009	IFNbeta-1b 250 *μ*g vs GA	1345	24	1.06 (0.97, 1.16)	1.05 (0.84, 1.31)
O'Connor (2) 2009	IFNbeta-1b 500 *μ*g vs GA	1347	24	0.97 (0.88, 1.06)	1.10 (0.88, 1.37)

AZA: azathioprine; GA: glatiramer acetate; IFN*β*: interferon-*β*; IVIg: IV immunoglobulin; PBO: placebo.

### 2.2 Gastric cancer

Oba et al.^10^ investigated disease-free survival (DFS) as a surrogate endpoint for the overall survival (OS) in patients with curative gastric cancer. The study included randomised clinical trials that compared adjuvant chemotherapy with surgery alone. DFS was defined as the time to cancer recurrence, second cancer or death from any cause. DFS and OS hazard ratios were estimated with five years of follow-up.

**Figure 2.**
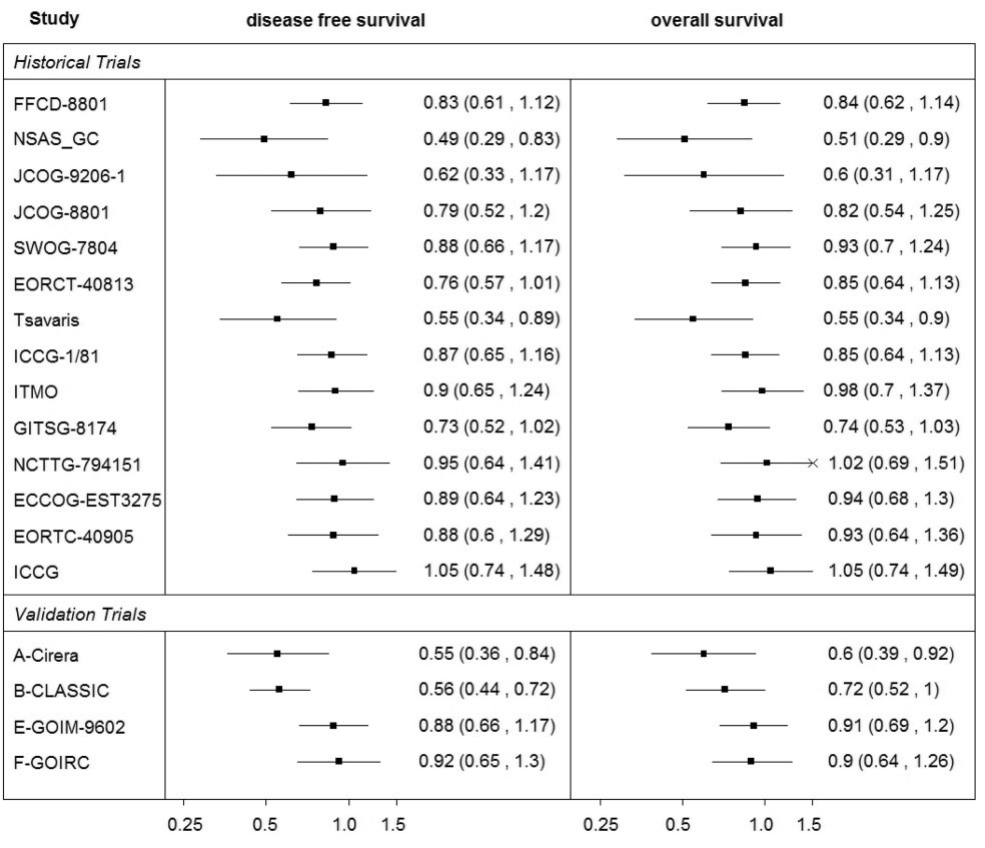
Summary of the ‘Oba data’.

We use data from Oba et al.^10^ as a second illustrative example to investigate the effect of the choice of a modelling technique and corresponding level of uncertainty on predictions. Data are presented in detail in Table 2 and graphically in Figure 2. We refer to these data as the ‘Oba data’ in the remainder of this paper. As in Oba et al, the studies are grouped as historical and validation studies. They are used in two sets of validation analyses, the cross-validation by taking out the effect on OS from one study at a time (this effect is predicted from DFS and the model based on the data on both outcomes from the remaining historical trials) and external validation where predictions are made for each of the validation trials using a model developed based on data from all the historical trials. As can be seen in Figure 2, the effects on DFS and OS have similar heterogeneity patterns between the studies suggesting a strong association between the effects on those outcomes.

**Table 2.** Studies in the ‘Oba data’ reporting the hazard ratio measured by the disease-free survival (DFS) and overall survival (OS).

Study	Number of patients		Follow-up	DFS	OS
	Chemotherapy	Surgery	(years)	HR (95% CI)	HR (95% CI)
*Historical trials*					
FFCD-8801	133	136	8.1	0.83 (0.61, 1.12)	0.84 (0.62, 1.14)
NSAS-GC	95	95	6.0	0.49 (0.29, 0.83)	0.51 (0.29, 0.90)
JCOG-9206-1	128	124	5.9	0.62 (0.33, 1.17)	0.60 (0.31, 1.17)
JCOG-8801	272	264	6.7	0.79 (0.52, 1.20)	0.82 (0.53, 1.26)
SWOG-7804	107	112	16.6	0.88 (0.66, 1.17)	0.93 (0.70, 1.24)
EORCT-40813	152	154	6.5	0.76 (0.57, 1.01)	0.85 (0.64, 1.13)
Tsavaris	44	44	4.9	0.55 (0.34, 0.89)	0.55 (0.33, 0.90)
ICCG-1/81	133	148	13	0.87 (0.65, 1.16)	0.85 (0.64, 1.13)
ITMO	135	136	6.2	0.90 (0.65, 1.24)	0.98 (0.70, 1.37)
GITSG-8174	90	88	12.1	0.73 (0.52, 1.02)	0.74 (0.53, 1.04)
NCTTG-794151	62	64	15.6	0.95 (0.64, 1.41)	1.02 (0.69, 1.51)
ECCOG-EST3275	91	89	16.5	0.89 (0.64, 1.23)	0.94 (0.68, 1.30)
EORTC-40905	103	103	7.0	0.88 (0.60, 1.29)	0.93 (0.64, 1.36)
ICCG	89	97	6.9	1.05 (0.74, 1.48)	1.05 (0.74, 1.49)
*Validation trials*					
A-Cirera	520	515	2.8	0.55 (0.36, 0.84)	0.60 (0.39, 0.93)
B-CLASSIC	76	72	3.1	0.56 (0.44, 0.72)	0.72 (0.52, 1.00)
E-GOIM-9602	112	113	5.0	0.88 (0.66, 1.17)	0.91 (0.69, 1.21)
F-GOIRC	130	128	6.1	0.92 (0.65, 1.30)	0.90 (0.64, 1.26)

Details of chemotherapy regimens can be found in the supplementary material of Oba et al.^10^

## 3 Methods for evaluating surrogate endpoints

In this section, the technical details of the meta-analytic models are listed with emphasis on the use of such methods to predict a treatment effect measured by a target outcome of interest from the effect measured by a surrogate endpoint. The prediction is based on the association between the treatment effects on the two outcomes evaluated by a model developed based on the data in a ‘training set’, usually data from historical studies available for both outcomes from which a model ‘learns’ the relationship between them.

The methods in a Bayesian framework are described in Sections 3.1 to 3.4. To investigate the impact of the choice of parameterisation on the uncertainty around the predicted effects, we start with the simplest model allowing for a minimum variability, the FEMR. We then increase the allowed variability in the model by the use of random effects meta-regression (REMR) and further by introducing bivariate meta-analytic models which allow for the measurement error of the treatment effect on the surrogate endpoint. Sensitivity analyses to prior distributions and the distributional assumptions are discussed in Sections 3.5 and 3.6, respectively. Some frequentist approaches are then discussed in Section 3.7. Strategies for the validation of surrogate endpoints and model comparison are described in Section 3.8.

### 3.1 Meta-regression

#### 3.1.1 Fixed-effects meta-regression

Linear or weighted regression models have been used to evaluate surrogate endpoints with regard to predictions,^7,8^ by including the treatment effect on a surrogate endpoint in the meta-analysis as a covariate. In the meta-analytic context, this approach can be described by the FEMR which in the Bayesian framework for normally distributed outcomes has the form

(1) $$Y_{2i}∼N(\mu_{2i},\sigma_{2i}^{2})\mu_{2i}=\lambda_{0}+\lambda_{1}Y_{1i}$$

with prior distributions $\lambda_{0},\lambda_{1}∼N(0.0,1000000)$. *Y*_1*i*_ and *Y*_2*i*_ are the estimates of the treatment effects on the surrogate and the final outcomes, respectively, with standard deviation *σ*_2*i*_ corresponding to the effect on the final outcome in each study *i*. The normally distributed effects *Y*_2*i*_ estimate underlying true effects *μ*_2*i*_. The intercept *λ*_0_ and slope *λ*_1_ define the relationship between the effects on the two outcomes.

Having estimated the parameters *λ*_0_ and *λ*_1_, this model can be used to predict the treatment effect on the target outcome based on the observed treatment effect on the surrogate endpoint. If for a new study *j*, the observed treatment effect on the surrogate outcome is *Y*_1*j*_ then, based on model (1), prediction is made using the regression equation

(2) μ^2j=λ0+λ1Y1j.

In this model, uncertainty around the predicted effect on the target outcome is related to the uncertainty around the intercept *λ*_0_, whereas the treatment effect on the surrogate endpoint is treated as a fixed covariate.

#### 3.1.2 Random effects meta-regression

A REMR model can be used to evaluate surrogate endpoints.^17^ The model allows for between-study variability by assuming that the treatment effects *Y*_2*i*_ estimate different underlying true effects *μ*_2*i*_ (regardless of the value of the covariate) in each study *i*. In a Bayesian framework, meta-regression can be formulated as in Sutton and Abrams^18^ in the following way using the random effects approach

(3) $$Y_{2i}∼N(\mu_{2i},\sigma_{2i}^{2})\mu_{2i}=\lambda_{0i}+\lambda_{1}Y_{1i}\lambda_{0i}∼N(\beta ,\psi 2)$$

where *Y*_1*i*_ are the summary measures of the treatment effect on the candidate surrogate outcome and *Y*_2*i*_ represent the summary measures of the treatment effect on the target outcome with corresponding standard deviations *σ*_2*i*_ from each study *i*. The normally distributed *Y*_2*i*_ are estimates of the underlying true effects *μ*_2*i*_. The *λ*_0*i*_ are the true effects at value zero of the treatment effect on the surrogate endpoint and they follow a common Normal distribution with mean *β* and standard deviation *ψ*, representing the between-study heterogeneity. The regression coefficient *λ*_1_ represents the relationship between the treatment effects on the target and the surrogate outcomes. In this Bayesian framework, all parameters are given prior distributions: $\beta ∼N(0.0,1000),\lambda_{1}∼N(0.0,1000000)$ and ψ∼N(0,100)I(0,) (a half-normal distribution truncated at zero).

The prediction can be made by

(4) μ^2j=λ0j+λ1Y1j,

where *λ*_0*j*_ is obtained from the model, by the use of the Markov chain Monte Carlo (MCMC) simulation, with data that include the new study, but the target outcome is coded as missing (NA in WinBUGS).

An alternative approach is also possible by centring the values of the effect on the surrogate, *Y*_1*i*_. In this case, the interpretation would change and the intercept would represent the true treatment effect on the final outcome at the average value of the effect on the surrogate endpoint. This approach could have an advantage when external information is available to construct an informative prior distribution to be placed on the intercept. Also, the centring of the effect on the surrogate may help to reduce the autocorrelation when conducting the MCMC simulation. However, for the purpose of predicting the effect for a new study, which is central to the evaluation of surrogate endpoints, the effect would have to be ‘un-centred’.

WinBUGS code corresponding to this model is included in Appendix 1.1.

### 3.2 Meta-analysis by Daniels and Hughes

In a model proposed by Daniels and Hughes,^6^ the estimates of the treatment effects measured by the surrogate endpoint *Y*_1*i*_ and the target outcome *Y*_2*i*_ are assumed to come from a bivariate normal distribution and they estimate the underlying true effects on the surrogate and target outcomes *μ*_1*i*_ and *μ*_2*i*_, respectively, from each study *i* with corresponding within-study standard deviations *σ*_1*i*_ and *σ*_2*i*_ and within-study correlation *ρ*_*wi*_

(5) $$(Y_{1i}Y_{2i})∼\text{MVN}((\mu_{1i}\mu_{2i}),(\sigma_{1i}^{2}\sigma_{1i}\sigma_{2i}\rho_{\mathrm{wi}}\sigma_{1i}\sigma_{2i}\rho_{\mathrm{wi}}\sigma_{2i}^{2}))\mu_{2i}|\mu_{1i}∼N(\lambda_{0}+\lambda_{1}\mu_{1i},\psi 2),$$

where the underlying true effects *μ*_1*i*_ measured by the surrogate endpoint are assumed to be fixed effects and to have a linear relationship with the true effect on the target outcome *μ*_2*i*_. Prior distributions are given to all parameters: $\mu_{1i}∼N(0,1000),\lambda_{0}∼N(0.0,1000),\lambda_{1}∼$ N(0.0,1000),ψ∼N(0,100)I(0,).

In this model, estimates of the treatment effects on both the target as well as the surrogate endpoints are treated as response variables and therefore the uncertainty around the treatment effect on the surrogate outcome is taken into account in this model. If for a study *j* the observed treatment effect on the surrogate outcome is *Y*_1*j*_, then the treatment effect on the target outcome *Y*_2*j*_ can be predicted from the model by assuming that this outcome is missing at random. By assuming that the two effects are correlated and follow a common bivariate distribution, the missing effect (on the target outcome in this case) is estimated automatically by the MCMC simulation, from the model which takes into account the correlation between the effects on the two outcomes. WinBUGS code for this model is listed in Appendix 1.2.

### 3.3 Bivariate random effects meta-analysis (BRMA)

Bivariate meta-analytic methods have been proposed for joint modelling of correlated outcomes^12,19^ and included approaches in a Bayesian framework.^20,21^ BRMA is discussed here in the form described by van Houwelingen et al.^12^ and Riley et al.,^13^ where estimates of treatment effect on both outcomes *Y*_1*i*_ and *Y*_2*i*_ are assumed to be normally distributed

(6) $$(Y_{1i}Y_{2i})∼\text{MVN}((\mu_{1i}\mu_{2i}),\Sigma_{i}),\;\Sigma_{i}=(\sigma_{1i}^{2}\sigma_{1i}\sigma_{2i}\rho_{\mathrm{wi}}\sigma_{1i}\sigma_{2i}\rho_{\mathrm{wi}}\sigma_{2i}^{2})$$

(7) $$(\mu_{1i}\mu_{2i})∼\text{MVN}((\beta_{1}\beta_{2}),T),\;T=(\tau_{1}^{2}\tau_{1}\tau_{2}\rho_{b}\tau_{1}\tau_{2}\rho_{b}\tau_{2}^{2}).$$

In this model, the treatment effect on the surrogate endpoint *Y*_1*i*_ and the treatment effect on the target outcome *Y*_2*i*_ are assumed to estimate the correlated true effects *μ*_1*i*_ and *μ*_2*i*_ with corresponding within-study variances $\sigma_{1i}^{2}$ and $\sigma_{2i}^{2}$ of the estimates and the within-study correlation *ρ*_*wi*_ between them. These true study-level effects follow a bivariate normal distribution with means $(\beta_{1},\beta_{2})$, between-study variances $\tau_{1}^{2}$ and $\tau_{2}^{2}$ and a between-study correlation *ρ*_*b*_ in this hierarchical framework. Equation (6) represents the within-study model and equation (7) is the between-study model. To implement the model in the Bayesian framework, prior distributions are placed on the between-study covariance matrix using the inverse Wishart distribution T-1∼Wishart((1001),3) where the degrees of freedom parameter was set to 3 (the dimension of the matrix plus 1) to induce a uniform prior distribution for the between-study correlation *ρ*_*b*_.^22^ Non-informative prior distributions are placed on the within-study correlations using uniform distributions $\rho_{\mathrm{wi}}∼U(-1,1)$ and on the mean effects $\beta_{1,2}∼N(0,10000)$.

As in the model (5) by Daniels and Hughes, the treatment effect on the target outcome in a study *j* can be predicted from the treatment effect on the surrogate endpoint observed by this study, by assuming that the effect on the target outcome is missing at random and assuming exchangeability of the treatment effects. In contrast to model (5), the BRMA model allows an estimation of the pooled effects measured by both outcomes (rather than only the pooled effect of the target endpoint in equation (5) which is only possible when centring the effect on the surrogate outcome on the mean). Although the ability to estimate the pooled effect does not impact on the validation process, it can be advantageous when modelling treatment effects on surrogate and target outcomes jointly to combine all available evidence in the assessment of the effectiveness. However, to make it possible, stronger distributional assumptions about the true effects are made in this model in comparison with model (5). WinBUGS code for this model is listed in Appendix 1.3.

### 3.4 BRMA in product normal formulation (BRMA PNF)

The BRMA models (6) and (7) can be parameterised in an alternative form where instead of placing a prior distribution on the between-study covariance matrix as a whole, the between-study model (7) is represented in the PNF^14,23^ (a product of univariate conditional normal distributions), whereas the within-study model remains the same

(8) $$(Y_{1i}Y_{2i})∼\text{MVN}((\mu_{1i}\mu_{2i}),\Sigma_{i}),\;\Sigma_{i}=(\sigma_{1i}^{2}\sigma_{1i}\sigma_{2i}\rho_{\mathrm{wi}}\sigma_{1i}\sigma_{2i}\rho_{\mathrm{wi}}\sigma_{2i}^{2})$$

(9) $$\{\mu_{1i}∼N(\eta_{1},\psi_{1}^{2})\mu_{2i}|\mu_{1i}∼N(\eta_{2i},\psi_{2}^{2})\eta_{2i}=\lambda_{0}+\lambda_{1}\mu_{1i}.$$

As for the BRMA model, *Y*_1*i*_ and *Y*_2*i*_ are the estimates of the treatment effects measured by the surrogate and target endpoints, respectively, and the *μ*_1*i*_ and *μ*_2*i*_ are the true effects in the population which are correlated and modelled here through a linear relationship. Prior distributions are placed on the following parameters: $\rho_{\mathrm{wi}}∼U(-1,1),\lambda_{0}∼N(0.0,1000),$ $\eta_{1}∼N(0.0,1000),\psi_{1}∼N(0,100)I(0,),\psi_{2}∼N(0,100)I(0,),\rho_{b}∼U(-1,1)$. The between-study variances are $\tau_{1}^{2}=\psi_{1}^{2}$ and $\tau_{2}^{2}=\psi_{2}^{2}+\lambda_{21}^{2}\psi_{1}^{2}$ and hence the implied prior distribution is placed on $\lambda_{1}=\frac{\psi_{2}}{\psi_{1}}\frac{\rho_{b}}{\sqrt{1-\left(\rho_{b}\right)2}}$.^14^

The PNF provides better control over the prior distributions placed on specific parameters of the model (compared to BRMA with Wishart prior distribution), helping to ensure that they are non-informative when this is required or allowing for informative prior distributions, based on external evidence, to be placed directly on the desirable parameters of the model.^14^ WinBUGS code corresponding to this model is included in Appendix 1.4.

### 3.5 Sensitivity analysis: Prior distributions

When investigating the impact of parameterisation and the related uncertainty on the precision of the predicted estimates, we carried out sensitivity analysis using a range of prior distributions for the heterogeneity parameters (*ψ* in meta-regression and model by Daniels and Hughes and $\psi_{1,2}$ in BRMA (PNF)). The following distributions were included:

- Prior I: ψ∼N(0,100)I(0,)
- Prior II: ψ∼N(0,10)I(0,)
- Prior III: $\frac{1}{\psi 2}∼\mathrm{Gamma}(0.001,0.001)$
- Prior IV: ψ∼Uniform(0,2).

Other examples of non-informative prior distributions can be found in the simulation study by Lambert et al.^24^ Sensitivity analysis was also carried out to investigate the impact of the choice of the parameters of the inverse Wishart prior distribution on the implied prior distributions for the heterogeneity parameters (while maintaining the implied uniform prior distribution on the between-study correlation). Wishart prior distributions with the following parameters were tested:

- Wishart A: T-1∼Wishart((1001),3)
- Wishart B: T-1∼Wishart((0.1000.1),3).

**Figure 3.**
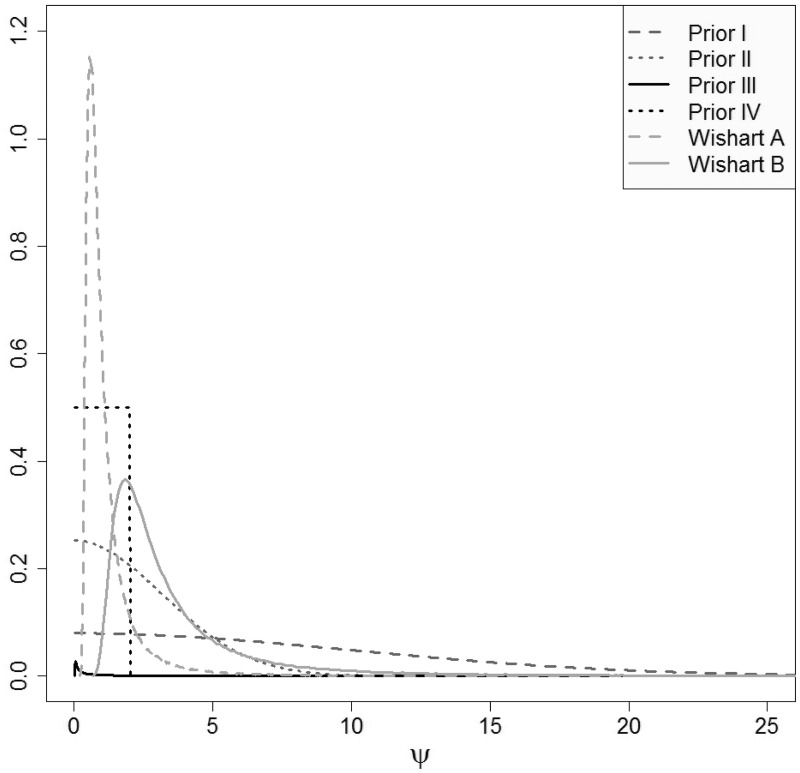
Prior distributions for the standard deviations used in the sensitivity analysis.

Figure 3 shows the prior distributions for the standard deviations overlayed (distributions I, II and IV used directly and distributions obtained from priors III, Wishart A and B by transformation on the standard deviation scale). Prior distributions I–III have large variances and hence are non-informative. The uniform prior distribution IV is locally non-informative on the scale of the modelled data. The implied prior distributions on the standard deviations obtained from the Wishart distributions placed on the between-study precision matrix are both quite informative (as mentioned above, the corresponding implied distribution on the between-study correlation is uniform on the range of values between –1 and 1).

### 3.6 Sensitivity analysis: Relaxing the normality assumption

The methods considered here are models with random effects to reflect the assumption that the modelled treatment effects are different between the studies. The differences in the effects may be due to the varying populations, different treatments under investigation in those studies or perhaps heterogeneity in the definitions of the outcomes.^25^ Typically, the normal distribution of the between-study random effects is assumed to reflect the similarity of the effects. The assumption that the true treatment effects on both outcomes (such as log relative risk and log rate ratio for the example in RRMS or log hazard ratio on OS and DFS in gastric cancer) are normally distributed may, however, not always be reasonable. When dealing with departures from normality of the modelled data, this assumption can lead to limitations of modelling and restricted inferences.^26^ For example, as discussed by Marshall and Spiegelhalter, inadequate use of normality assumption about the random effects may lead to ‘overshrinkage’ of the true effects and hence to misleading inferences.^27^

One way of relaxing this assumption is to use a *t*-distribution as recommended, for example, by Lee and Thompson^26^ or Smith et al.^28^ In contrast to the normal distribution, the *t*-distribution gives more weight in the tails which is more likely to be better at modelling extreme effects such as outlying observations.^27^ We apply the *t*-distribution to the random effect in the BRMA model by adapting its PNF form. In the product of *t*-distributions formulation (PTDF), the between-study model can be formulated as

(10) $$\{\mu_{1i}∼t(\eta_{1},\nu_{1},\mathrm{df})\mu_{2i}|\mu_{1i}∼t(\eta_{2i},\nu_{2},\mathrm{df})\eta_{2i}=\lambda_{0}+\lambda_{1}\mu_{1i}.$$

with prior distributions placed on the parameters, $\lambda_{0},\lambda_{1}∼N(0.0,1000)$ and $\eta_{1}∼N(0.0,1000)$. Placing non-informative prior distributions on the between-study standard deviations corresponding to the true effects *μ*_1*i*_ and *μ*_2*i*_, $\tau_{1}∼N(0,100)I(0,)$ and $\tau_{2}∼N(0,100)I(0,)$ gives implied prior distributions on the corresponding parameters, $\nu_{1}=(\tau_{1}^{2*}(\mathrm{df}-2))/\mathrm{df}$ and $\nu_{2}=(\tau_{2}^{2*}(\mathrm{df}-2))/\mathrm{df}$. WinBUGS code corresponding to this model is included in Appendix 1.5.

### 3.7 Frequentist approaches

The above models for evaluation of surrogate endpoints differ in the way they take into account the uncertainty around the model parameters. The Bayesian framework gives a flexible environment for modelling of uncertainty. Some of the models, however, can be also implemented in a frequentist approach using software such as, for example, Stata. To compare the different degrees of uncertainty allowed by different frequentist models, two models are compared here: the meta-regression and the bivariate meta-analysis.

#### 3.7.1 Meta-regression

Suppose *Y*_1*i*_ is the estimate of the treatment effect on the candidate surrogate outcome and *Y*_2*i*_ represents the estimate of the treatment effect on the target outcome with corresponding within-study variance *v*_2*i*_ in study *i* (i=1,…,n). In the frequentist framework, meta-regression for the association between the effects on the surrogate and the target endpoints can be written following the formulation by Sharp^29^ in the following form

(11) $$Y_{2}∼N(Y_{1}\lambda ,V)$$

where $Y_{2}=(Y_{21},\ldots ,Y_{2n})T$ is the n×1 vector of the treatment effect on the final outcome and $Y_{1}$ is the n×2 design matrix with *i*th row $(1,Y_{1i}),\lambda =(\lambda_{0},\lambda_{1})T$ is the vector of parameters and **V** is a diagonal *n* × *n* variance matrix with *i*th diagonal element $v_{2i}+\tau 2$, where the *τ*^2^ represents the between-study variability for the random effects model. Maximum likelihood methods are used to estimate the parameters λ and *τ*^2^ and in Stata this can be achieved by using the command metareg. The predictions are made using the linear predictor, and in Stata using the post-estimation command predict.

#### 3.7.2 Bivariate meta-analysis

As in the Bayesian framework, the random effects bivariate meta-analysis can be described in the hierarchical framework

(12) $$(Y_{1i}Y_{2i})∼\text{MVN}((\mu_{1i}\mu_{2i}),{-20\%\Sigma }_{i}),\;{-20\%\Sigma }_{i}=(\sigma_{1i}^{2}\sigma_{1i}\sigma_{2i}\rho_{\mathrm{wi}}\sigma_{1i}\sigma_{2i}\rho_{\mathrm{wi}}\sigma_{2i}^{2})$$

(13) $$(\mu_{1i}\mu_{2i})∼\text{MVN}((\beta_{1}\beta_{2}),T),\;T=(\tau_{1}^{2}\tau_{1}\tau_{2}\rho_{b}\tau_{1}\tau_{2}\rho_{b}\tau_{2}^{2}).$$

with the treatment effect on the surrogate endpoint *Y*_1*i*_ and the treatment effect on the target outcome *Y*_2*i*_ in each study *i* and corresponding within-study variances of the estimates $\sigma_{1i}^{2}$ and $\sigma_{2i}^{2}$ and the within-study correlation *ρ*_*wi*_ between them. The correlated true effects *μ*_1*i*_ and *μ*_2*i*_ follow bivariate normal distribution with means $(\beta_{1},\beta_{2})$, between-study variances $\tau_{1}^{2}$ and $\tau_{2}^{2}$ and a between-study correlation *ρ*_*b*_. In Stata, the model can be implemented using the command mvmeta.^30^ In the Bayesian framework, the predicted estimates for the final endpoint assumed missing at random are obtained from a MCMC simulation. Here, we obtain the estimate of the true effect on the final outcome for study *j* as follows

(14) $$E(\mu_{j}|Y_{j},\beta ,T)=\beta +({-20\%\Sigma }_{j}+T)-1\;T\;(Y_{j}-\beta )$$

(15) $$\mathrm{var}(\mu_{j}|Y_{j},\beta ,T)=({-20\%\Sigma }_{j}+T)-1\;T\;{-20\%\Sigma }_{j},$$

where $Y_{j},\mu_{j}$ and β are two-dimensional vectors and ${-20\%\Sigma }_{j}$ and **T** are 2 × 2 matrices.

Stata code for the model predictions using the meta-regression and the BRMA is included in Appendix 1.6.

### 3.8 Cross-validation procedure and model comparison

Evaluation of surrogate endpoints on the study level, assessing whether the treatment effect on the final outcome can be predicted from the treatment effect on the surrogate endpoint, can be carried out by the take-one-out approach in the cross-validation procedure, as described by Daniels and Hughes.^6^ This procedure aims to establish goodness of fit of the meta-analytic prediction model. In each study the effect on the final outcome is assumed unknown (in one study at a time) and it is then predicted from the effect on the surrogate endpoint, conditional on the data on the treatment effects on both outcomes from the remaining studies and the parameters of the model.

Ultimately we want to draw inferences about predicting the true effect on the final outcome *μ*_2*j*_ in a future study *j*. However, in a real data scenario (as opposed to simulated data) we do not know what the true effect is. Hence for the purpose of the cross-validation, we predict the ‘observed estimate’ Y^2j. For this purpose, we assume *σ*_2*j*_ known and hence effectively only the true effect *μ*_2*j*_ is predicted. We then check if the observed value of *Y*_2*j*_ falls within the predicted interval of Y^2j with the standard deviation equal to σ2j2+var(μ^2j|Y1j,σ1j,Y1(-j),Y2(-j)), where $Y_{1(-j)}$ and $Y_{2(-j)}$ denote the data from the remaining studies without the validation study *j*.

To investigate the impact of the uncertainty on predictions, we compare the models with respect to the predicted intervals. To compare how the choice of parameterisation affects the uncertainty of predictions, we compare the widths of the intervals of the predicted Y^2j and predicted true effects μ^2j across the models. To do so, we summarise the ratios wY^2j/wY2j of the widths of the intervals for Y^2j to the widths of the intervals for *Y*_2*j*_ to investigate how this varies across the models and the ratios wμ^2jCM/wμ^2jFEMR of the widths of the predicted true effects μ^2j from each current model (CM) to the width of the predicted interval for μ^2j obtained from the FEMR.

## 4 Results

### 4.1 Results from Bayesian models: multiple sclerosis

To compare the models, in the first instance the estimates of the pooled effects on both outcomes, the relapse rate ratio and the disability progression rate ratio, were obtained from all the models. Due to the large heterogeneity of the control arm between the studies (and the fact that an intervention which is a control arm in one study may be an experimental arm in the other) only placebo-controlled studies were included in this particular estimation. The inclusion of all studies would not give clinically interpretable results and in order to combine evidence from all the trials in a sensible way, a network meta-analysis would need to be conducted which is beyond the scope of this paper. Note that the whole data set (including both placebo- and active-controlled trials) is used for the remaining analyses that focus on the predictions for the purpose of evaluation of surrogate endpoints. The results shown in Table 3 are for the comparison of models only. Both forms of BRMA allowed for the estimation of the pooled effect of both outcomes, in contrast to meta-regression and model by Daniels and Hughes which allowed estimation of the pooled effect on the disability progression only. The pooled effect measured by the surrogate endpoint, relapse rate ratio, was the same using both forms of BRMA. The point estimate of the pooled effect measured by the target endpoint, the disability progression rate ratio, was the same for all models but obtained with different precisions from different models. The largest uncertainty around the estimate was obtained from the BRMA model with the Wishart A prior distribution placed on the between-study precision matrix. Effectiveness estimates of the highest precision were obtained from the meta-regression and the model by Daniels and Hughes. Relatively high precision of the pooled effect was also obtained from BRMA PNF.

**Table 3.** Summary results for placebo-controlled studies for the treatment effects on the risk of disability progression and relapse rate ratio.

	Relapse incidence rate ratio			Disability relative risk			
Model	Mean	95% CrI	$\tau_{1}$^a^ (sd)	Mean	95% CrI	*ψ*_2_ (sd)	*τ*_2_ (sd)
REMR				0.75^b^	[0.67; 0.84]	0.07 (0.06)	
D&H^c^				0.75^b^	[0.66; 0.84]	0.07 (0.06)	
BRMA	0.57	[0.44; 0.72]	0.44 (0.09)	0.75	[0.58; 0.95]		0.38 (0.09)
BRMA PNF	0.57	[0.46; 0.70]	0.36 (0.09)	0.75	[0.65; 0.87]	0.10 (0.06)	0.15 (0.08)

a $\psi_{1}=\tau_{1}$ in BRMA PNF.

b Obtained by centring the effects on surrogate endpoint on the mean. ^c^D&H refers to the model by Daniels & Hughes.

All four models were then applied to make predictions in a cross-validation procedure. The treatment effect on the final outcome (disease progression rate ratio) in the 25 studies was assumed unknown (in one study at a time which in that case became a validation study) and then predicted from the surrogate endpoint (relapse rate ratio) by each model.

Table 4 lists all the predictions made by all of the models for all of the studies (using prior distribution I for the heterogeneity parameter and Wishart A for the between-study precision matrix). For most studies, all models gave predicted Y^2j with intervals containing the corresponding observed estimates, except for one study by Durelli for which only the interval obtained from BRMA with Wishart prior B contained the observed estimate of the treatment effect. Most intervals obtained from BRMA with Wishart prior A were largely inflated apart from the interval in study by Miligan which was the smallest study with largest intervals for the treatment effects on both outcomes.

**Table 4.** Predictions obtained from all models for all studies in the ‘Sormani data’.

	Disability progression rate ratio, mean (95% CrI)				
	Paty (1)	Paty (2)	Miligan	Johnson	Jacobs/Simon
Observed	1.00 (0.67, 1.49)	0.71 (0.45, 1.12)	1.14 (0.26, 5.03)	0.88 (0.57, 1.35)	0.63 (0.38, 1.05)
Meta-regression (FE)	0.99 (0.66, 1.48)	0.84 (0.53, 1.33)	0.93 (0.21, 4.11)	0.87 (0.56, 1.35)	0.85 (0.51, 1.42)
Meta-regression (RE)	0.99 (0.64, 1.53)	0.84 (0.52, 1.35)	0.92 (0.21, 4.13)	0.87 (0.54, 1.38)	0.85 (0.50, 1.45)
Daniels & Hughes	0.99 (0.63, 1.54)	0.84 (0.51, 1.37)	0.93 (0.20, 4.31)	0.87 (0.54, 1.41)	0.85 (0.50, 1.46)
BRMA (Wishart)	1.00 (0.47, 2.13)	0.81 (0.39, 1.68)	0.83 (0.16, 4.29)	0.81 (0.36, 1.82)	0.82 (0.36, 1.87)
BRMA (PNF)	0.97 (0.60, 1.57)	0.83 (0.49, 1.40)	0.86 (0.19, 3.95)	0.86 (0.52, 1.42)	0.83 (0.47, 1.48)
	Fazekas	Millefiorini	Achiron	Li (1)	Li (2)
Observed	0.70 (0.36, 1.35)	0.19 (0.05, 0.79)	0.82 (0.19, 3.50)	0.81 (0.61, 1.08)	0.73 (0.54, 0.99)
Meta-regression (FE)	0.66 (0.34, 1.29)	0.61 (0.14, 2.55)	0.63 (0.15, 2.69)	0.87 (0.65, 1.17)	0.86 (0.63, 1.17)
Meta-regression (RE)	0.65 (0.33, 1.30)	0.60 (0.14, 2.53)	0.62 (0.14, 2.67)	0.87 (0.62, 1.21)	0.85 (0.60, 1.20)
Daniels & Hughes	0.65 (0.32, 1.32)	0.60 (0.14, 2.60)	0.62 (0.14, 2.73)	0.87 (0.62, 1.22)	0.86 (0.60, 1.23)
BRMA (Wishart)	0.70 (0.28, 1.76)	0.65 (0.14, 3.16)	0.64 (0.13, 3.16)	0.85 (0.43, 1.68)	0.84 (0.39, 1.79)
BRMA (PNF)	0.67 (0.33, 1.38)	0.65 (0.15, 2.81)	0.67 (0.15, 2.97)	0.86 (0.58, 1.25)	0.84 (0.57, 1.24)
	Clanet	Durelli	Baumhackl	Polman	Rudick
Observed	1.00 (0.83, 1.20)	0.43 (0.24, 0.78)	1.08 (0.74, 1.57)	0.59 (0.46, 0.75)	0.79 (0.65, 0.96)
Meta-regression (FE)	1.08 (0.87, 1.34)	0.88 (0.48, 1.59)*	0.94 (0.64, 1.39)	0.58 (0.43, 0.78)	0.66 (0.53, 0.83)
Meta-regression (RE)	1.08 (0.82, 1.43)	0.87 (0.48, 1.61)*	0.94 (0.62, 1.43)	0.57 (0.40, 0.80)	0.66 (0.51, 0.86)
Daniels & Hughes	1.10 (0.84, 1.44)	0.88 (0.47, 1.64)*	0.94 (0.60, 1.46)	0.57 (0.39, 0.82)	0.66 (0.51, 0.87)
BRMA (Wishart)	1.04 (0.60, 1.79)	0.84 (0.35, 2.01)	0.91 (0.42, 1.95)	0.56 (0.27, 1.15)	0.69 (0.30, 1.59)
BRMA (PNF)	1.07 (0.77, 1.48)	0.85 (0.45, 1.61)*	0.91 (0.58, 1.44)	0.57 (0.37, 0.88)	0.67 (0.48, 0.94)
	Coles (1)	Coles (2)	Mikol	Comi (1)	Comi (2)
Observed	0.35 (0.16, 0.74)	0.38 (0.19, 0.77)	1.34 (0.88, 2.06)	0.69 (0.52, 0.93)	0.73 (0.55, 0.97)
Meta-regression (FE)	0.58 (0.27, 1.26)	0.49 (0.24, 1.01)	1.03 (0.66, 1.60)	0.66 (0.48, 0.91)	0.69 (0.51, 0.93)
Meta-regression (RE)	0.58 (0.26, 1.26)	0.48 (0.23, 1.01)	1.03 (0.65, 1.63)	0.65 (0.46, 0.93)	0.68 (0.48, 0.95)
Daniels & Hughes	0.58 (0.26, 1.30)	0.49 (0.23, 1.05)	1.04 (0.64, 1.69)	0.64(0.42, 0.99)	0.67 (0.45, 1.00)
BRMA (Wishart)	0.64 (0.23, 1.75)	0.60 (0.22, 1.58)	0.92 (0.43, 1.97)	0.59 (0.28, 1.22)	0.71 (0.30, 1.67)
BRMA (PNF)	0.63 (0.28, 1.41)	0.55 (0.25, 1.21)	0.97 (0.59, 1.59)	0.68 (0.45, 1.04)	0.69 (0.46, 1.05)
	Havrdova (1)	Havrdova (2)	Sorensen	O'Connor (1)	O'Connor (2)
Observed	1.23 (0.58, 2.62)	1.04 (0.48, 2.27)	0.64 (0.32, 1.28)	1.05 (0.84, 1.31)	1.10 (0.88, 1.37)
Meta-regression (FE)	0.96 (0.45, 2.05)	0.86 (0.39, 1.88)	0.63 (0.31, 1.27)	1.06 (0.83, 1.37)	1.00 (0.78, 1.27)
Meta-regression (RE)	0.96 (0.44, 2.07)	0.86 (0.39, 1.89)	0.62 (0.30, 1.27)	1.07 (0.78, 1.45)	1.00 (0.75, 1.34)
Daniels & Hughes	0.96 (0.43, 2.10)	0.86 (0.38, 1.92)	0.62 (0.29, 1.31)	1.06 (0.79, 1.42)	0.99 (0.75, 1.32)
BRMA (Wishart)	0.93 (0.34, 2.51)	0.81 (0.30, 2.19)	0.63 (0.24, 1.65)	0.84 (0.43, 1.65)	0.95 (0.48, 1.87)
BRMA (PNF)	0.93 (0.42, 2.07)	0.84 (0.37, 1.92)	0.66 (0.31, 1.42)	1.01 (0.70, 1.47)	0.98 (0.68, 1.39)

The discrepancies between the observed and predicted values were obtained for all studies (by taking the absolute difference between the observed estimate of the treatment effect and the predicted effect) and summarised in Table 5, which also summarises the degree of uncertainty around the predicted estimate compared to the uncertainty around the observed value (by calculating the ratio wY^2j/wY2j of the length of the 95% predicted interval to the length of the 95% confidence interval of the observed estimate, shown in the second to last column of the table). Note that the intervals of the predicted Y^2j were inflated compared to those corresponding to the observed effects *Y*_2*j*_ due to the additional between-study variability. To compare uncertainty of predicted true effects across models, ratio wμ^2jCM/wμ^2jFEMR of the length of the 95% credible interval around μ^2j obtained from the CM to the length of that interval from the FEMR was calculated and presented in the last column of Table 5.

**Table 5.** Results of the comparison of the models for predicting the treatment effect on disability progression from the treatment effect on relapse rate.

		Absolute discrepancy	wY^2j/wY2j	wμ^2jCM/wμ^2jFEMR
Model	Prior	Median (range)	Median (range)	Median (range)
FEMR		0.16 (0.01, 1.16)	1.02 (1.00, 1.21)	
REMR	I	0.15 (0.01, 1.15)	1.07 (1.00, 1.54)	1.96 (1.36, 2.56)
REMR	II	0.16 (0.01, 1.15)	1.07 (1.01, 1.52)	1.95 (1.34, 2.53)
REMR	III	0.15 (0.01, 1.15)	1.07 (1.01, 1.51)	1.91 (1.36, 2.43)
REMR	IV	0.16 (0.01, 1.15)	1.07 (1.01, 1.51)	1.93 (1.37, 2.58)
Daniels & Hughes	I	0.16 (0.01, 1.15)	1.11 (1.02, 1.50)	2.44 (1.65, 5.14)
Daniels & Hughes	II	0.17 (0.02, 1.16)	1.11 (1.02, 1.56)	2.28 (1.62, 5.78)
Daniels & Hughes	III	0.16 (0.01, 1.15)	1.11 (1.02, 1.59)	2.43 (1.61, 5.15)
Daniels & Hughes	IV	0.16 (0.01, 1.16)	1.11 (1.02, 1.45)	2.43 (1.51, 5.11)
BRMA PNF	I	0.14 (0.02, 1.23)	1.16 (1.02, 1.83)	2.95 (1.95, 4.85)
BRMA PNF	II	0.16 (0.01, 1.23)	1.18 (1.02, 1.73)	2.88 (2.02, 4.68)
BRMA PNF	III	0.15 (0.00, 1.23)	1.11 (1.02, 1.52)	2.26 (1.45, 4.48)
BRMA PNF	IV	0.15 (0.01, 1.24)	1.17 (1.02, 1.86)	2.90 (1.74, 4.92)
BRMA	Wishart A	0.16 (0.00, 1.24)	1.78 (1.10, 4.27)	7.00 (3.48, 10.07)
BRMA	Wishart B	0.13 (0.00, 1.23)	1.23 (1.03, 1.95)	3.28 (2.09, 5.60)

CM: current model in each row.

The accuracy of predictions for the point estimate was similar across models, but the uncertainty around the predicted effects varied depending on the parameterisation. Using the meta-regression equation (2) the effect on the target outcome was predicted with much increased precision compared to other models. For example, when using prior distribution I the interval for the predicted true effect μ^2j from the REMR was almost twice as wide (on log relative risk scale) compared to the interval obtained from the FEMR. The results obtained from the models by Daniels and Hughes and BRMA PNF were much more conservative with moderately reduced precision (with intervals, respectively, 2.44 and 2.95 times wider than those obtained from the FEMR). When applying the BRMA model with a Wishart prior distribution, the results were sensitive to the parameters of the prior distribution. In the case of Wishart A distribution with identity matrix, the predicted intervals were largely inflated (most likely due to implied prior distributions on the between-study variances not being suitably non-informative). Using the Wishart B prior distribution led to predictions comparable to those obtained from BRMA PNF with slightly more inflated intervals. The use of the REMR approach, as in equation (4), resulted in increased uncertainty around the predicted effect on the disability progression (compared to predictions obtained when using the FEMR approach) of similar magnitude to the results obtained from models by Daniels and Hughes and BRMA PNF. This uncertainty in the predictions obtained from REMR can be related to the number of studies in the set or the level of the between-study heterogeneity and hence precision can be gained when using a larger set of studies. The same scenario applies to some extent to other models as well. This is mostly the case for the model by Daniels and Hughes which has a form similar to the REMR, but in addition the uncertainty in this model is related to the uncertainty around the effect on the surrogate endpoint, while this is not the case when using meta-regression which includes the effect on surrogate endpoint as a fixed covariate. Similarly, BRMA PNF gives predictions with uncertainty related to both the size and heterogeneity of the data set (as well as the uncertainty around the effect on the surrogate outcome); however, perhaps less so because of strong distributional assumptions about the between-study heterogeneity which leads to a greater effect of ‘borrowing of strength’ across the studies and the outcomes. Sensitivity analysis in relation to the choice of the prior distribution placed on the standard deviations (*ψ* in the meta-regression and model by Daniels and Huhges, and *ψ*_1_ and *ψ*_2_ in the BRMA PNF) was carried out as described in Section 3.5. The sensitivity analyses using prior distributions I–IV gave very similar results as can be seen in Table 5. As mentioned above, predictions were sensitive to the parameters of the Wishart prior distribution.

The results suggest that prediction of true effects obtained from the FEMR (and potentially also REMR) can be overly optimistic and artificially precise, likely with intervals not containing the true value, due to underestimated between-study variability and the measurement error corresponding to the treatment effect on the surrogate endpoint (relapse rate ratio in this case). However, the success of the prediction may also be affected by the strong assumptions about the distribution of the data made in the models, such as for example exchangeability assumption in BRMA PNF. To investigate this further, a simulation study was conducted which is presented in Section 5.

#### 4.1.1 Discussion of the results for RRMS

Based on our results we cannot conclude that relapse rate is a good surrogate for disability progression as the prediction did not give good results for all of the studies (it failed for the study by Durelli using all methods apart from the BRMA with Wishart prior (A) which largely inflated the variance of predictions). The study by Durelli differs from the rest of the set in that the effect on the disability progression is much larger than the effect on the relapse rate, with the ratio of the relative effects on those outcomes (the effect on progression to the effect on relapse) equal to 0.6. In most of the remaining studies, this ratio is usually higher than 1.0 (it ranges between 0.94 and 2.16) owing to the fact that disability progression is a longer term outcome and the effect measured on this outcome at the same follow-up time as the effect on the relapse rate will be less due to relatively few events occurring for this outcome on this time scale. The only other study with that ratio below one was the study by Millefiorini, with the ratio of 0.56. The cross-validation did not fail for this study likely because it is a small study with estimates of the treatment effects on both outcomes having large variances (included in the predicted intervals for the cross-validation).

In the Millefiorini study, the patients were relatively young compared to the other studies with a relatively high baseline disability score which can explain the extreme treatment effect on disability of the mitoxantrone relative to the effect of the placebo. The baseline relapse rate was more representative of other studies and hence the effect on this outcome was less extreme (albeit still substantial). There does not seem to be anything, however, in the population of the study by Durelli that would explain the opposite relationship in the magnitude of the effects on the two outcomes. The patients were slightly older compared to other studies and the average baseline disability score was relatively low. This may suggest that the treatment effect on annualised relapse rate may not be a perfect predictor of the effect on the disability progression rate. However, the predictions overwhelmingly worked for the remaining studies which would encourage further research. Note that the effect on the final outcome in the data set investigated here is measured at the same time point as the effect on the surrogate endpoint. Since the disability progression is considered a long-term endpoint, when measured early it is measured with a relatively large uncertainty due to low number of events. Further research is required to establish whether the relapse rate is a good surrogate endpoint and in particular an early marker of disability progression. Such further research should include disability progression reported later compared to relapse rate, but potentially also consider both outcomes on alternative scales such as the hazard ratio for the time to disability progression. Sormani et al. already point out the limitations of using the summary data alone to evaluate the surrogate outcomes. To properly establish the surrogacy, outcomes on an individual level need to be investigated ideally based on data from all of the clinical trials.

### 4.2 Results from Bayesian models: Gastric cancer

As in the case of RRMS, in the first instance pooled effects were obtained using the historical trials data set to compare the models. The data were then used to perform the cross-validation of the surrogate endpoints. ‘Oba data’ also included another group of studies, the validation trials, which were then used for external validation. Pooled effects obtained from all of the models are shown in Table 6 for comparison. As noted in the previous section on RRMS, only the two forms of BRMA allowed for the estimation of the pooled treatment effects on both outcomes. The pooled effect measured by the surrogate endpoint, DFS, had higher uncertainty in BRMA Wishart (A) model compared to BRMA PNF model. The point estimate of the pooled treatment effect measured by the target endpoint, OS, was similar for all models. Moreover, all models gave estimates with similar precisions except for the BRMA model with inverse Wishart (A) prior which resulted in estimates with a remarkably higher uncertainty.

**Table 6.** Summary results for treatment effect on overall survival and disease-free survival.

	Disease-free survival			Overall survival			
Model	Mean	95% CrI	$\tau_{1}(\mathrm{sd})$ ^a^	Mean	95% CrI	*ψ*_2_ (sd)	*τ*_2_ (sd)
REMR				0.81^b^	[0.73; 0.90]	0.05 (0.04)	
D&H^c^				0.82^b^	[0.74; 0.91]	0.05 (0.04)	
BRMA	0.84	[0.67; 1.02]	0.35 (0.08)	0.80	[0.64; 0.98]		0.35 (0.07)
BRMA PNF	0.87	[0.79; 0.95]	0.05 (0.04)	0.84	[0.76; 0.91]	0.04 (0.04)	0.05 (0.05)

a $\psi_{1}=\tau_{1}$ in BRMA PNF.

b Obtained by centring the effects on surrogate endpoint on the mean. ^c^D&H refers to the model by Daniels & Hughes.

When applying the four models to cross-validation, the effect on OS in the historical studies was assumed unknown (in one study at a time which in that case became a validation study) and then predicted from the effect on DFS by each model. The predicted effects on OS with corresponding intervals obtained for each historical study from each model are presented in Table 7 along with the predictions obtained for the validation studies. For one study (B-CLASSIC), the predicted effects on OS obtained from both meta-regression models were statistically significant while the observed effect was only borderline significant (predictions marked in bold font). This could be interpreted to be due to the fact that the effect on DFS is likely to be measured with higher precision due to a larger number of events observed on this outcome compared to OS. Therefore, when predicting the treatment effect on OS from the effect on DFS, higher precision can be expected. However, it occurred only when using meta-regression, not when using other methods, and hence was likely due to underestimated uncertainty by not including measurement error corresponding to the treatment effect on DFS when making the predictions. As in the RRMS example, most intervals obtained from BRMA with Wishart prior A were largely inflated.

**Table 7.** Predictions obtained from all models for all studies in the ‘Oba data’.

Overall survival, mean (95% CrI)					
	**Historical trials**				
	FFCD-8801	NSAS-GC	JCOG-9206-1	JCOG-8801	SWOG-7804
Observed	0.84 (0.62, 1.14)	0.51 (0.29, 0.90)	0.60 (0.31, 1.18)	0.82 (0.54, 1.27)	0.93 (0.70, 1.24)
Meta-regression	0.87 (0.63, 1.19)	0.50 (0.25, 1.01)	0.65 (0.32, 1.30)	0.82 (0.53, 1.27)	0.91 (0.67, 1.24)
Meta-regression 2	0.86 (0.61, 1.23)	0.50 (0.24, 1.03)	0.64 (0.31, 1.31)	0.82 (0.52, 1.30)	0.91 (0.65, 1.28)
Daniels & Hughes	0.86 (0.55, 1.33)	0.62 (0.30, 1.31)	0.73 (0.32, 1.67)	0.85 (0.48, 1.51)	0.90 (0.60, 1.33)
BRMA (Wishart)	0.90 (0.45, 1.80)	0.84 (0.32, 2.17)	0.82 (0.31, 2.16)	0.72 (0.30, 1.74)	0.84 (0.39, 1.82)
BRMA (PNF)	0.87 (0.61, 1.25)	0.87 (0.48, 1.57)	0.87 (0.43, 1.72)	0.88 (0.56, 1.38)	0.86 (0.60, 1.21)
	EORTC-40813	Tsavaris	ICCG-1/81	ITMO	GITSG-8174

Observed	0.85 (0.64, 1.14)	0.55 (0.33, 0.89)	0.85 (0.64, 1.13)	0.98 (0.70, 1.37)	0.74 (0.53, 1.04)
Meta-regression	0.78 (0.57, 1.06)	0.58 (0.32, 1.03)	0.91 (0.67, 1.24)	0.93 (0.65, 1.33)	0.76 (0.53, 1.09)
Meta-regression 2	0.78 (0.56, 1.10)	0.57 (0.31, 1.05)	0.91 (0.65, 1.28)	0.93 (0.64, 1.36)	0.76 (0.52, 1.12)
Daniels & Hughes	0.79 (0.52, 1.19)	0.67 (0.35, 1.32)	0.91 (0.59, 1.40)	0.92 (0.59, 1.44)	0.78 (0.49, 1.25)
BRMA (Wishart)	0.81 (0.41, 1.63)	0.81 (0.33, 1.97)	0.88 (0.39, 1.97)	0.87 (0.35, 2.16)	0.83 (0.38, 1.80)
BRMA (PNF)	0.86 (0.62, 1.21)	0.87 (0.51, 1.46)	0.87 (0.62, 1.22)	0.87 (0.61, 1.24)	0.87 (0.60, 1.27)
	NCTTG-794151	ECCOG-EST3275	EORTC-40905	ICCG	

Observed	1.02 (0.69, 1.51)	0.94 (0.68, 1.30)	0.93 (0.64, 1.37)	1.05 (0.74, 1.49)	
Meta-regression	0.99 (0.65, 1.49)	0.92 (0.66, 1.30)	0.91 (0.62, 1.36)	1.11 (0.74, 1.66)	
Meta-regression 2	0.99 (0.64, 1.53)	0.93 (0.64, 1.34)	0.91 (0.60, 1.39)	1.11 (0.72, 1.71)	
Daniels & Hughes	0.95 (0.55, 1.64)	0.91 (0.57, 1.44)	0.89 (0.53, 1.50)	0.99 (0.62, 1.59)	
BRMA (Wishart)	0.87 (0.38, 2.02)	0.80 (0.38, 1.70)	0.92 (0.42, 2.01)	0.89 (0.42, 1.92)	
BRMA (PNF)	0.86 (0.56, 1.32)	0.86 (0.59, 1.24)	0.87 (0.56, 1.33)	0.86 (0.59, 1.25)	

**Validation trials**					
	A-cirera	B-CLASSIC	E-GOIM-9602	F-GOIRC	

Observed	0.60 (0.39, 0.93)	0.72 (0.52, 1.00)	0.91 (0.69, 1.21)	0.90 (0.64, 1.26)	
Meta-regression	0.57 (0.34, 0.94)	**0.58 (0.38, 0.88)**	0.92 (0.68, 1.23)	0.96 (0.67, 1.37)	
Meta-regression 2	0.57 (0.34, 0.96)	**0.58 (0.37, 0.89)**	0.92 (0.66, 1.27)	0.96 (0.65, 1.40)	
Daniels & Hughes	0.62 (0.33, 1.16)	0.62 (0.38, 1.02)	0.90 (0.61, 1.32)	0.93 (0.59, 1.48)	
BRMA (Wishart)	0.79 (0.32, 1.94)	0.70 (0.32, 1.55)	0.84 (0.41 1.73)	0.80 (0.34, 1.84)	
BRMA (PNF)	0.86 (0.54, 1.36)	0.80 (0.53, 1.20)	0.87 (0.63, 1.20)	0.87 (0.60, 1.26)	

Discrepancies between observed and predicted estimates of the treatment effect on OS, summarised by the absolute difference and the ratio of the width of the predicted interval wY^2j to the width of the interval corresponding to the observed estimate $w_{Y_{2j}}$, are presented in Table 8 (column three and second to last, respectively). The absolute discrepancies were highest when using bivariate meta-analysis (both PNF and Wishart), which may suggest that the exchangeability assumption about the true treatment effects was too strong for these data. As expected, the predicted intervals of Y^2j are inflated (compared to the intervals of *Y*_2*j*_) due to the between-study variability in addition to the sampling variance. Intervals from the model by Daniels and Hughes were wider compared to those obtained from the REMR, likely due to the measurement error around the treatment effect on the surrogate endpoint (DFS in this case) taken into account in this model. This is also seen in the ratios of the widths of the predicted intervals of the true effects obtained from each model wμ^2jCM to the width of the predicted interval wμ^2jFEMR obtained from the FEMR (last column in Table 8) which suggests that predictive intervals obtained from the FEMR may be underestimated due to the ignored uncertainty. This is further investigated by a simulation study in Section 5. The results are in agreement with those obtained for the RRMS example in Section 4.1. However, unlike in the example in RRMS, the predicted intervals obtained from BRMA PNF are narrower compared to those obtained from the model by Daniels and Hughes. The inclusion of measurement error around the treatment effect on the surrogate endpoint is balanced by the ‘borrowing of strength’ across studies by the exchangeability assumption which in this case is likely to cause ‘overshrinkage’, as discussed in Section 3.6. This is consistent with the absolute discrepancies being larger when using the BRMA models compared to, for example, the model by Daniels and Hughes which does not make the assumption of the exchangeability. As already noted in Section 4.1, this issue is explored by the simulation in Section 5. The BRMA with inverse Wishart prior distribution gave much inflated intervals for Wishart A, but not for Wishart B prior distribution which confirms the sensitivity of the results to the parameters of the Wishart distribution as already observed in the RRMS example. Sensitivity analyses in relation to the choice of the prior distribution placed on the standard deviations (*ψ* in the meta-regression and model by Daniels and Huhges, and *ψ*_1_ and *ψ*_2_ in the BRMA PNF) were carried out as described in Section 3.5. The sensitivity analyses using prior distributions I–IV gave very similar results as can be seen in Table 8.

**Table 8.** Results of the comparison of the models for predicting the treatment effect on OS from the treatment effect on DFS.

		Absolute discrepancy	wY^2j/wY2j	wμ^2jCM/wμ^2jFEMR
Model	Prior	Median (range)	Median (range)	Median (range)
FEMR		0.03 (0.00, 0.09)	1.06 (1.03, 1.23)	
REMR	I	0.03 (0.00, 0.08)	1.15 (1.07, 1.27)	1.59 (1.11, 1.76)
REMR	II	0.03 (0.00, 0.09)	1.15 (1.07, 1.27)	1.60 (1.10, 1.78)
REMR	III	0.03 (0.00, 0.09)	1.15 (1.07, 1.27)	1.61 (1.15, 1.77)
REMR	IV	0.03 (0.00, 0.09)	1.15 (1.07, 1.26)	1.59 (1.08, 1.73)
Daniels & Hughes	I	0.06 (0.02, 0.20)	1.38 (1.23, 1.52)	2.70 (1.15, 3.89)
Daniels & Hughes	II	0.05 (0.03, 0.18)	1.39 (1.24, 1.48)	2.58 (1.38, 3.79)
Daniels & Hughes	III	0.05 (0.01, 0.17)	1.36 (1.28, 1.43)	2.68 (1.15, 3.96)
Daniels & Hughes	IV	0.06 (0.01, 0.21)	1.37 (1.19, 1.46)	2.64 (1.25, 3.13)
BRMA PNF	I	0.11 (0.01, 0.53)	1.10 (1.03, 1.22)	1.46 (0.47, 1.95)
BRMA PNF	II	0.11 (0.01, 0.53)	1.11 (1.03, 1.18)	1.57 (0.43, 1.83)
BRMA PNF	III	0.11 (0.01, 0.52)	1.14 (1.05, 1.24)	1.75 (0.51, 2.07)
BRMA PNF	IV	0.10 (0.01, 0.53)	1.10 (1.03, 1.18)	1.48 (0.47, 1.81)
BRMA	Wishart A	0.12 (0.01, 0.49)	2.24 (1.44, 2.83)	5.97 (2.17, 8.44)
BRMA	Wishart B	0.11 (0.01, 0.49)	1.37 (1.11, 1.55)	2.85 (0.89, 3.60)

#### 4.2.1 Discussion of the results for gastric cancer

The cross-validation of the predictions of the treatment effect on the OS from the effect on the DFS confirmed the results of Oba et al. recommending that DFS is a good surrogate endpoint for OS in patients with curable gastric cancer. One of the limitations of this case study was the absence of any delay between the measurement of the effect on the surrogate endpoint and the final outcome. Ideally, one would be interested in establishing whether DFS measured early could be used to predict long-term OS in the new trials. Sensitivity analysis conducted by Oba et al. was inconclusive whether or not the treatment effect on DFS measured as early as at two years of follow-up can be a good predictor of the treatment effect on OS estimated with five years of follow-up.^10^

### 4.3 Results of sensitivity analysis with t-distribution

As discussed in Section 3.6, sensitivity analysis was carried out to investigate the effect of the distributional assumptions by using the *t*-distribution on the random effect. Tables 9 and 10 show results of applying the PTDF model to the ‘Sormani data’ for the example in RRMS. Sensitivity analyses were carried out by varying the degrees of freedom parameter using values 4, 15 and 30. The results are presented alongside those obtained from BRMA PNF with comparable prior distributions (the same prior distributions as for PTDF in Section 3.6). The models with the *t*-distribution gave very similar results across all values for the degrees of freedom parameter and also when compared to the results obtained from BRMA PNF. The only noticeable, but still very small, difference was for the model with *df* = 4 where the uncertainty around the pooled effect on relapse rate was slightly higher and the estimate of the heterogeneity parameter for the effect on this endpoint was also higher and with higher uncertainty (results in Table 9). All models gave very similar discrepancies in terms of the absolute difference and the ratios of the widths of the intervals comparing predicted and observed effects, wY^2j/wY2j, and the widths of the intervals of the predicted true effects from PTDF models compared to the predicted intervals from BRMA PNF, wμ^2jPTDF/wμ^2jPNF as shown in Table 10. Consistently with the results in Table 9, the intervals obtained from PTDF model with *df* = 4 were slightly wider compared to those obtained from BRMA PNF and PTDF with *df* = 15 or 30.

**Table 9.** Summary results for placebo-controlled studies for the treatment effects on the risk of disability progression and the relapse rate ratio in RRMS, using models with *t*-distributions and BRMA PNF for comparison.

	Relapse incidence rate ratio			Disability relative risk		
Model	Mean (SD)	95% CrI	*ψ*_1_	Mean (SD)	95% CrI	*ψ*_2_
BRMA PNF	0.57 (0.06)	[0.46; 0.70]	0.37 (0.09)	0.75 (0.05)	[0.67; 0.86]	0.07 (0.06)
BRMA PTDF (4 df)	0.58 (0.07)	[0.46; 0.72]	0.47 (0.14)	0.75 (0.05)	[0.66; 0.85]	0.08 (0.07)
BRMA PTDF (15 df)	0.57 (0.06)	[0.45; 0.71]	0.39 (0.10)	0.75 (0.05)	[0.66; 0.85]	0.08 (0.06)
BRMA PTDF (30 df)	0.57 (0.06)	[0.45; 0.71]	0.38 (0.10)	0.75 (0.05)	[0.67; 0.85]	0.07 (0.06)

**Table 10.** Results of the comparison of the models for predicting the treatment effect on the risk of disability progression from the treatment effect on relapse rate in RRMS, using models with *t*-distributions and BRMA PNF for comparison.

	Absolute discrepancy	wY^2j/wY2j	wμ^2jPTDF/wμ^2jPNF
Model	Median (range)	Median (range)	Median (range)
BRMA PNF	0.16 (0.01, 1.22)	1.10 (1.02, 1.58)	
BRMA PTDF (4 df)	0.16 (0.01, 1.22)	1.12 (1.02, 1.64)	1.04 (0.97, 1.15)
BRMA PTDF (15 df)	0.16 (0.01, 1.21)	1.10 (1.02, 1.57)	1.01 (0.96, 1.06)
BRMA PTDF (30 df)	0.16 (0.00, 1.22)	1.11 (1.02, 1.55)	1.01 (0.97, 1.08)

As it can be seen in Tables 11 and 12, the results from the models applied to the ‘Oba data’ for the example in gastric cancer were also very similar across the range of values of the degrees of freedom. Median interval ratio comparing the predicted to the observed effects was highest for *df* = 4, but still comparable with the results corresponding to other parameters and those from BRMA PNF. Predicted intervals of the true effects from PTDF model with *df* = 4 were wider than those obtained from BRMA PNF, with the median ratio of the widths wμ^2jPTDF/wμ^2jPNF=1.06, but less so when *df* = 15 or 30 as expected. All predictions for both data sets are included in Tables A 2.1 and A 2.2 in Appendix 2. The results were similar to those obtained from the BRMA PNF model leading to the same conclusions.

**Table 11.** Summary results for treatment effects on overall survival and disease-free survival RRMS, using models with t-distributions and BRMA PNF for comparison.

	Disease-free survival			Overall survival		
Model	Mean (SD)	95% CrI	*ψ*_1_	Mean (SD)	95% CrI	*ψ*_2_
BRMA PNF	0.83 (0.04)	[0.76; 0.92]	0.03 (0.04)	0.87 (0.04)	[0.79; 0.95]	0.05 (0.04)
BRMA PTDF (4 df)	0.83 (0.04)	[0.76; 0.91]	0.03 (0.05)	0.87 (0.04)	[0.79; 0.94]	0.05 (0.05)
BRMA PTDF (15 df)	0.83 (0.04)	[0.76; 0.90]	0.03 (0.05)	0.86 (0.04)	[0.79; 0.94]	0.05 (0.04)
BRMA PTDF (30 df)	0.83 (0.04)	[0.76; 0.90]	0.03 (0.04)	0.86 (0.04)	[0.79; 0.94]	0.05 (0.04)

**Table 12.** Results of the comparison of the models for predicting treatment effect on OS from treatment effect on DFS, using models with t-distributions and BRMA PNF for comparison.

	Absolute discrepancy	wY^2j/wY2j	wμ^2jPTDF/wμ^2jPNF
Model	Median (range)	Median (range)	Median (range)
BRMA PNF	0.11 (0.02, 0.52)	1.18 (1.05, 1.27)	
BRMA PTDF (4 df)	0.11 (0.02, 0.52)	1.21 (1.06, 1.34)	1.06 (0.98, 1.19)
BRMA PTDF (15 df)	0.11 (0.01, 0.52)	1.17 (1.04, 1.27)	1.00 (0.93, 1.10)
BRMA PTDF (30 df)	0.11 (0.01, 0.52)	1.17 (1.05, 1.29)	1.01 (0.92, 1.08)

### 4.4 Results from the frequentist models

Table 13 shows the discrepancies between the predicted and observed values of the effect on the final outcome (in terms of the median absolute difference between the estimates and the median ratio of the width of the 95% predicted interval to the width of the 95% confidence interval corresponding to the observed effect) for the ‘Sormani data’ and the ‘Oba data’. The absolute discrepancies are comparable with those obtained from the Bayesian models. The effect of the model choice on the uncertainty of predictions is represented by the ratios wμ^2jBRMA/wμ^2jFEMR of the width of the predicted intervals for the true effects obtained from the BRMA model to the interval obtained from the FEMR. The differences in the width of the predicted intervals between the models are consistent with the conclusions from the Bayesian analysis; the predictive interval is inflated when using BRMA (with the median ratio wμ^2jBRMA/wμ^2jFEMR=1.69 in the RRMS example and wμ^2jBRMA/wμ^2jFEMR=1.41 for gastric cancer data) which allows the inclusion of the uncertainty on the effects on both outcomes alongside all other parameters.

**Table 13.** Results of the comparison of the frequentist models for predicting the treatment effect on disability progression from treatment effect on relapse in RRMS and the treatment effect on OS from the treatment effect on DFS in gastric cancer.

	Absolute discrepancy	wY^2j/wY2j	wμ^2jBRMA/wμ^2jFEMR
Model	Median (range)	Median (range)	Median (range)
*RRMS*			
FEMR	0.16 (0.01, 1.16)	1.02 (1.00, 1.21)	
BRMA	0.16 (0.00, 1.24)	1.06 (1.06, 1.12)	1.69 (0.52, 4.90)
*Gastric cancer*			
FEMR	0.04 (0.00, 0.09)	1.08 (1.03, 1.25)	
BRMA	0.10 (0.02, 0.52)	1.10 (1.01, 1.15)	1.41 (0.20, 1.71)

Tables A 3.1 and A 3.2 in Appendix 3 list predicted estimates on the final outcome (disability progression in RRMS and OS in gastric cancer). When using meta-regression, the predictions were obtained with reduced intervals (compared to the intervals corresponding to those obtained from BRMA). As in the Bayesian analysis, predicted interval for one study (B-CLASSIC) in the example in gastric cancer indicated significant effect (numbers in bold) when using FEMR (but not BRMA) while the observed effect was only borderline significant. Note that in the frequentist analysis, the within-study correlation is fixed (instead of the prior distributions in the Bayesian analysis). The results in Tables 13, A 3.1 and A 3.2 were obtained from models with *ρ*_*wi*_ = 0.5. Sensitivity analysis using correlations $\rho_{\mathrm{wi}}=0,\;0.25,\;0.75$ gave very similar results.

## 5 Simulation

The models considered in this paper allow for different level of uncertainty on the parameters and use different degree of distributional assumptions, both of which can impact on the accuracy of predictions. The models by Daniels and Hughes and the BRMA PNF seemed to predict the treatment effect on the target outcome equally well, giving conservative predictions (in comparison with meta-regression) because uncertainty around all the model parameters is taken into account, but not with overly inflated intervals. The two models, however, use a different degree of distributional assumptions. Considering, for example, a scenario where a new study may measure a treatment effect much larger compared to the effect observed in the historical studies (training set), the assumption in the BRMA PNF (about the true effects measured by both outcomes coming from a common distribution) may be too strong. Sensitivity to this assumption along with the performance of all the models is tested here by a simulation.

### 5.1 Methods

To carry out the simulation, data were simulated for both the validation studies as well as the ‘training set’ to ensure the control over the distributional assumptions of the data (the ‘Sormani data’ did not satisfy the assumption of normality well). Simulation of the validation data and the training set data was conducted using the BRMA PNF model (8) and (9) in a number of scenarios where the mean of the effect in the validation set is shifted by *δ* relative to the mean of the training set

(16) $$(Y_{1i}Y_{2i})∼\text{MVN}((\mu_{1i}\mu_{2i}),\Sigma_{i}),\;\Sigma_{i}=(\sigma_{1i}^{2}\sigma_{1i}\sigma_{2i}\rho_{\mathrm{wi}}\sigma_{1i}\sigma_{2i}\rho_{\mathrm{wi}}\sigma_{2i}^{2})$$

(17) $$\{\mu_{1i}∼N(\eta_{1}+\delta ,\psi_{1}^{2})\mu_{2i}|\mu_{1i}∼N(\eta_{2i},\psi_{2}^{2})\eta_{2i}=\lambda_{0}+\lambda_{1}\mu_{1i}.$$

using a range of values of *δ*s: 0, *ψ*_1_, $2\psi_{1},3\psi_{1}$ and 5*ψ*_1_. The higher the *δ* the more different the ‘new study’ is with respect to the training set. Parameters for the simulation were obtained by fitting the model to the ‘Sormani data’ which gave $\psi_{1}=0.36,\psi_{2}=0.15$, $\eta_{1}=-0.5253,\lambda_{0}=0.01$ and *λ*_1_ = 0.4793. The within-study correlations *ρ*_*wi*_ were sampled from a uniform distribution with limits obtained from the confidence interval of the mean of estimated within-study correlations, $\rho_{\mathrm{wi}}∼U(-0.11,0.186)$. The within-study variances were generated by sampling the corresponding precisions (inverse variances) from the gamma distribution; $\sigma_{1i}=1/P_{1i}$ and $\sigma_{2i}=1/P_{2i},P_{1i}∼\Gamma (\alpha_{1},\theta_{1})$, $P_{2i}∼\Gamma (\alpha_{2},\theta_{2})$, where *α*_1_ and *α*_2_ are the shape parameters and *θ*_1_ and *θ*_2_ the scale parameters, which were obtained using the method of moments: $E(P_{1,2})=\alpha_{1,2}/\xi_{1,2}$, $V(P_{1,2})=\alpha_{1,2}/\xi_{1,2}^{2}$, where $\xi_{1,2}=1/\theta_{1,2}$ is a rate parameter. By summarising the inverse variances from the ‘Sormani data’, the following parameters were obtained: $E(P_{1})=112.6$, $E(P_{2})=32.2,V(P_{1})=11172.49,V(P_{2})=1062.76$, giving the following shape and rate parameters: *α*_1_ = 1.13, $\xi_{1}=0.01,\alpha_{2}=0.97$ and $\xi_{2}=0.03$. Because of the structure of the gamma distribution, some of the simulated precisions were very close to zero, resulting in very large variances. This led to some problems with the estimation. To overcome this issue, a constraint was placed on the simulated value of the precision by discarding the precisions resulting in variances larger than 3 (this number was taken as an arbitrary cut off, large enough to be much larger than the variances in the ‘Sormani data’ and hence including all plausible variances in the population but small enough not to produce problems with the estimation). The number of participants in each study was drawn from a uniform distribution with limits 25 and 100 (giving sample sizes of the studies comparable to those in the ‘Sormani data’).

Each model was fitted by adding a validation study to the training set (one at a time) assuming the effect on the target outcome (disability progression) unknown (coded as NA), which was then predicted by each model from the effect on the relapse rate given for this study. The predicted true effect μ^2 was compared with the simulated ‘observed’ true effect *μ*_2_ by checking if the credible interval of the predicted effect on the target outcome contained the observed mean effect. The whole process was repeated 1000 times and the percentage of predicted outcomes whose credible intervals covered the observed value was reported as the average performance of the credible interval of the model. The R code used to simulate the data is included in Appendix 1.7.

### 5.2 Results

Table 14 lists the average performances of predicted credible interval for each model and for the range of values of *δ*. Moving the ‘new study’ (validation study) away from the ‘training set’ (by increasing the *δ*) resulted in reduced performance of the BRMA PNF, while the model by Daniels and Hughes preformed better (due to the lack of the strong distributional assumption of exchangeability of the true effects made in the BRMA PNF). Performance of BRMA PTDF remained unchanged due to the *t*-distribution being better at modelling extreme effects, as noted in Section 3.6.

**Table 14.** Comparison of the performance of the models in terms of the coverage of the predictive interval.

	Average performance of credible interval				
Model	*δ* = 0	$\delta =1\psi_{1}$	$\delta =2\psi_{1}$	$\delta =3\psi_{1}$	$\delta =5\psi_{1}$
FEMR	39%	41%	49%	56%	60%
REMR	95%	93%	93%	92%	90%
Daniels & Hughes	95%	94%	95%	94%	93%
BRMA (Wishart)	97%	96%	96%	94%	90%
BRMA (PNF)	96%	95%	93%	91%	85%
BRMA PTDF (4 df)	96%	95%	96%	95%	95%

BRMA model with the Wishart prior distribution showed slightly too large performance for *δ* = 0 which was related to the overly inflated predictive intervals. FEMR performed least well due to the artificially reduced uncertainty by ignoring the estimation error of the treatment effect on the surrogate endpoint. In this case, the performance seems to increase with the validation set moving away from the training set which is due to the predicted interval expanding as we move further away from the data, as in linear regression.

## 6 Discussion

When investigating endpoints as candidate surrogate outcomes, a careful choice of the meta-analytical approach has to be made. The level of uncertainty taken into account by the model can impact on the precision of the predictions of the true effect on the final outcome μ^2j from the effect on the surrogate endpoint. Models underestimating uncertainty, such as FEMR can lead to overly precise predictions of the treatment effect on the final outcome in a new study. Reduced uncertainty around predicted treatment effect on a target endpoint may give the illusion that this is a desirable effect of a larger number of events measured on the shorter term surrogate endpoint, whilst in fact this may be due to ignoring uncertainty and in the case of some models between-study variability. Models underestimating the uncertainty of available evidence may lead to over-optimistic predictions which can then have an effect on decisions made based on such predictions, i.e. underpowered clinical trials or unrealistic cost-effectiveness outcomes.

In the models by Daniels and Hughes and BRMAs, the treatment effect on the surrogate endpoint is treated as a response variable and its uncertainty is taken into account in the model in contrast to the meta-regression model where the effect on the surrogate was a fixed covariate. BRMA with the inverse Wishart prior distribution on the between-study covariance matrix seems an unreliable approach because it does not allow the analyst to easily control the prior distributions on the specific elements of the covariance matrix. Results obtained from the model are sensitive to the parameters of the Wishart distribution. For example, setting parameters of the Wishart distribution that lead to a desirable non-informative uniform distribution induced on the between-study correlation can give undesirably informative prior distributions for the between-study standard deviations, which depending on the parameters can lead to inflated intervals for pooled or predicted estimates. For the illustrative examples considered here, this led to the inflation of the uncertainty around the predicted target outcome when using the Wishart distribution with the identity matrix and degrees of freedom equal to three. The BRMA PNF and Daniels–Hughes models predict the target outcome better, but make different distributional assumptions that need to be considered when making a choice between these methods. While the Daniels–Hughes model makes less strong distributional assumptions and may perform better when the new study differs from the historical data in the meta-analysis data set, the BRMA PNF has an advantage over it by allowing the estimation of pooled effects for both outcomes when combining data reported on one or both of them, which can be desirable when the pooled effectiveness estimates are of interest as is often the case in HTA. In circumstances when the distributional assumptions are plausible in BRMA PNF, this model has an additional advantage of allowing the analyst to incorporate external information (based on external evidence or expert opinions) in the form of informative prior distributions with the potential to reduce uncertainty around the estimate of interest.^14,31^

When using meta-analytic methods to predict the treatment effect on a target outcome of interest from the treatment effect measured by a surrogate endpoint, modelling assumptions need to be considered alongside the uncertainty, particularly around the surrogate endpoint. While Bayesian methods allow for a great flexibility in modelling uncertainty, the frequentist methods have also been used to account for the uncertainty around the surrogate endpoint by using an error-in-variables linear regression model,^9,10^ which is an alternative for analysts with a preference for a frequentist approach. We have illustrated the importance of uncertainty by using frequentist methods of meta-regression and bivariate meta-analysis.

In this paper, to investigate the impact of uncertainty on predictions, we focused on a number of different parameterisations of normally distributed effects. The assumption of normality is not always reasonable and when it is not, alternative approaches need to be investigated. In our further work (to be published elsewhere) we investigate, for example, modelling of relapse rate using a Poisson distribution and the relative risk of disability progression by assuming that outcomes come from Binomial distribution. Meta-analytic methods using these type of outcomes have already been proposed, for example by Stijnen et al. who propose binomial-normal and Poisson-normal bivariate model (with binomial or Poisson distributions for the within-study variability).^32^ We have investigated the normality assumption on the random effect by sensitivity analysis where we replaced the normal distribution with the *t*-distribution. This approach has the limitation of only improving the modelling when there are more data in the tails (such as outlying observations) that a normal distribution would not capture properly. If the distribution of the data is, for example, bimodal or skewed, other approaches can be investigated such as a convolution of normal distributions^33^ or skewed *t*-distribution as proposed by Lee and Thompson.^26^ The issue of non-normality of the random effect has been discussed by Higgins et al.,^25^ who also review non-parametric alternatives of the meta-analytic methods that can be applied to the non-normally distributed effects (such as non-parametric maximum likelihood procedures^34–37^ and Bayesian semiparametric random-effects distributions based on Dirichlet process priors^38–40^). However, as Higgins et al. discuss, although the methods have the ability to incorporate outliers, they are not suitable for making predictions due to the unusual shape of the discrete distributions. As such, they are unlikely to be suitable for the purpose of evaluating surrogate endpoints where predictions are of crucial importance.

The methods discussed in this paper do not fully cover all aspects of the surrogate evaluation process. As already mentioned in Section 1, the individual level association between outcomes needs to be explored and to do so, individual patient data is required on a number (preferably all) of the studies included in the meta-analysis. Although this was beyond the scope of this paper, the availability of individual level data could help to model uncertainty. For example, individual data can be used to obtain the within-study correlation between the treatment effects. Daniels and Hughes have used individual level data from a subset of studies in their meta-analysis to obtain the correlation between the treatment effects by bootstrapping^6^ while Bujkiewicz et al. performed a double bootstrap analysis on individual level data from a single study to obtain the correlation between the treatment effects in the form of an empirical distribution.^14^ A range of methods for obtaining the within-study correlation from individual level data was explored by Riley et al. who used a joint linear regression for multiple continuous outcomes and bootstrapping methods for a range of other outcomes.^41^ The availability of individual level data can also be desirable when taking into account the information on covariates which in the aggregate form is subject to ecological bias. When investigating surrogacy, the inclusion of covariates could help explain some heterogeneity or explore the effect of baseline risk. Further research is required to explore the advantages of individual level data in modelling uncertainty and exploring the impact of covariates.

## Appendix 1

### Appendix 1.1. WinBUGS code: Random effects meta-regression

model{

for (i in 1:num) {

prec2[i]<-1/pow(se[i],2)

Y2[i] ∼ dnorm(mu2[i], prec2[i])

mu2[i]<-lambda0[i]+lambda1*(Y1[i]-mean(Y1[]))

lambda0[i] ∼dnorm(beta,prec1)

}

beta ∼dnorm(0.0, 0.001)

lambda1 ∼ dnorm(0.0,1.0E-6)

psi ∼dnorm(0,0.01)I(0,)

psi.sq<-psi*psi

prec1<-1/psi.sq

y2.uncent<-beta-lambda1*mean(X[])

mean2<-exp(beta)

new.y2<-y2.uncent+lambda1*new.log.y1

new.exp.y2<-exp(new.y2)

}

### Appendix 1.2. WinBUGS code: Daniels and Hughes model

model{

# within study precision matrices

for (i in 1:num) {

rho_w[i] ∼ dunif(-1,1)

prec_w[i,1:2,1:2]<-inverse(delta[i,1:2,1:2])

#covariance matrix for the j-th study

delta[i,1,1]<-var[i,1]/n[i,1]

delta[i,2,2]<-var[i,2]/n[i,2]

delta[i,1,2]<-sqrt(delta[i,1,1])*sqrt(delta[i,2,2])*rho_w[i]

delta[i,2,1]<-sqrt(delta[i,1,1])*sqrt(delta[i,2,2])*rho_w[i]

}

# Random effects model

for (i in 1:num) {

Y[i,1:2] ∼dmnorm(mu[i,1:2], prec_w[i,1:2,1:2])

mu[i,1] ∼dnorm(0,1.0E-3)

mu[i,2] ∼dnorm(edss[i],prec_dis)

edss[i]<-lambda0+lambda1*(mu[i,1] - mean(mu[,1]))

}

psi ∼dnorm(0,0.01)I(0,)

psi.sq<-psi*psi

prec_dis<-1/psi.sq

lambda0 ∼dnorm(0.0, 1.0E-3)

lambda1 ∼dnorm(0.0, 1.0E-3)

# estimates:

mean.log.dis<-lambda0

sd.log.dis<-sqrt(psi.sq)

mean.dis<-exp(mean.log.dis)

}

### Appendix 1.3. WinBUGS code: BRMA model with Wishart prior distribution

model{

#within study precision matrices

for (i in 1:num) {

rho_w[i] ∼dunif(-1,1)

prec_w[i,1:2,1:2]<-inverse(delta[i,1:2,1:2])

#covariance matrix for the j-th study

delta[i,1,1]<-var[i,1]/n[i,1]

delta[i,2,2]<-var[i,2]/n[i,2]

delta[i,1,2]<-sqrt(delta[i,1,1])*sqrt(delta[i,2,2])*rho_w[i]

delta[i,2,1]<-sqrt(delta[i,1,1])*sqrt(delta[i,2,2])*rho_w[i]

}

# Random effects model

for (i in 1:num) {

Y[i,1:2] ∼dmnorm(mu[i,1:2], prec_w[i,1:2,1:2])

mu[i,1:2] ∼dmnorm(beta[1:2],prec_b[1:2,1:2])

}

for (j in 1:2) {

beta[j] ∼dnorm(0.0,1.0E-4)

}

prec_b[1:2,1:2] ∼dwish(Q[,],3)

cov_b[1:2,1:2]<-inverse(prec_b[,])

# estimates:

mean.log.rel<-beta[1]

mean.log.dis<-beta[2]

sd.log.dis<-sqrt(cov_b[2,2])

sd.log.rel<-sqrt(cov_b[1,1])

corr.dis.rel<-cov_b[1,2]/(sd.log.dis*sd.log.rel)

psi.sq<-cov_b[2,2]*(1-corr.dis.rel*corr.dis.rel)

}

### Appendix 1.4. WinBUGS code: BRMA model in the product normal formulation

model{

# within study precision matrices

for (i in 1:num) {

rho_w[i] ∼dunif(-1,1)

prec_w[i,1:2,1:2]<-inverse(delta[i,1:2,1:2])

#covariance matrix for the j-th study

delta[i,1,1]<-var[i,1]/n[i,1]

delta[i,2,2]<-var[i,2]/n[i,2]

delta[i,1,2]<-sqrt(delta[i,1,1])*sqrt(delta[i,2,2])*rho_w[i]

delta[i,2,1]<-sqrt(delta[i,1,1])*sqrt(delta[i,2,2])*rho_w[i]

}

# Random effects model

for (i in 1:num) {

Y[i,1:2] ∼dmnorm(mu[i,1:2], prec_w[i,1:2,1:2])

*# product normal formulation for the between study part*:

mu[i,1] ∼dnorm(rel,prec_rel)

mu[i,2] ∼dnorm(edss[i],prec_dis)

edss[i]<-lambda0+lambda1*mu[i,1] }

rel ∼dnorm(0.0, 0.001)

gam_rel ∼dnorm(0,0.01)I(0,)

gam_dis ∼dnorm(0,0.01)I(0,)

gam_rel.sq<-gam_rel*gam_rel

gam_dis.sq<-gam_dis*gam_dis

prec_rel<-1/gam_rel.sq

prec_dis<-1/gam_dis.sq

lambda0 ∼dnorm(0.0, 1.0E-3)

# prior between study correlations:

corr.dis.rel ∼dunif(-1,1)

*# implied prior for lambda*

lambda1<-(gam_dis/gam_rel)*(corr.dis.rel/sqrt(1-corr.dis.rel*corr.dis.rel))

# estimates:

mean.log.rel<-rel

mean.log.dis<-lambda0 + lambda1 * mean.log.rel

sd.log.rel<-gam_rel

sd.log.dis<-sqrt(gam_dis.sq+gam_rel.sq*pow(lambda1,2))

mean.rel<-exp(mean.log.rel)

mean.dis<-exp(mean.log.dis)

}

### Appendix 1.5. WinBUGS code: BRMA model in the product of t-distributions formulation

model{

# within study precision matrices

for (i in 1:num) {

rho_w[i] ∼dunif(-1,1)

prec_w[i,1:2,1:2]<-inverse(delta[i,1:2,1:2])

#covariance matrix for the j-th study

delta[i,1,1]<-var[i,1]/n[i,1]

delta[i,2,2]<-var[i,2]/n[i,2]

delta[i,1,2]<-sqrt(delta[i,1,1])*sqrt(delta[i,2,2])*rho_w[i]

delta[i,2,1]<-sqrt(delta[i,1,1])*sqrt(delta[i,2,2])*rho_w[i]

}

# Random effects model

for (i in 1:num) {

Y[i,1:2] ∼dmnorm(mu[i,1:2], prec_w[i,1:2,1:2])

*# product of t-distributions formulation for the between study part*:

mu[i,1] ∼dt(rel,prec_rel,d)

mu[i,2] ∼dt(edss[i],prec_dis,d)

# edss[i]<-lambda0+lambda1*mu[i,1]

edss[i]<-lambda0+lambda1*(mu[i,1]- mean(mu[,1])) #when centered

}

rel ∼dnorm(0.0, 0.001)

gam_rel ∼dnorm(0,0.01)I(0,)

gam_dis ∼dnorm(0,0.01)I(0,)

gam_rel.sq<-gam_rel*gam_rel

gam_dis.sq<-gam_dis*gam_dis

prec_rel<-d / (gam_rel.sq * (d - 2))

prec_dis<-d / (gam_dis.sq * (d - 2))

lambda0 ∼dnorm(0.0, 1.0E-3)

lambda1 ∼dnorm(0.0, 1.0E-3)

# prior between study correlations:

corr.dis.rel ∼dunif(-1,1)

# estimates:

mean.log.rel<-rel

# mean.log.dis<-lambda0 + lambda1 * mean.log.rel

mean.log.dis<-lambda0 #when centered

mean.rel<-exp(mean.log.rel)

mean.dis<-exp(mean.log.dis)

}

### Appendix 1.6. Stata code for meta-regression and BRMA

Columns in the data contain treatment effects y1 and y2 (here on log scale) with corresponding standard errors se1 and se2.

forvalues k=1/25 {

use MSdata.dta, clear

gen b1=y1

gen b2=y2

gen se2n=se2

egen id=seq()

replace b2=. if id==‘k’

replace se2=. if id==‘k’

gen S11=se1^2

gen S22=se2^2

gen S12=se1*se2*0.5

replace b2 = 0 if b2==.

replace S22 = 10000 if S22==.

replace S12 = 0 if S12==.

*** mvmeta ***

mvmeta b S, mm keepmat(y S)

*** prediction ***

* set up

mat mu = e(b)

mat Sigma = e(Sigma)

* do calculations as matrices

forvalues i=1/25 {

mat eb‘i’ = mu + (y‘i’-mu) * syminv(S‘i’ + Sigma) * Sigma

mat vb‘i’ = Sigma * syminv(S‘i’ + Sigma) * S‘i’

}

* store as variables

forvalues r=1/2 {

qui gen eb‘r’=.

qui gen vb‘r’=.

forvalues i=1/25 {

qui replace eb‘r’ = eb‘i’[1,‘=‘r”] in ‘i’

qui replace vb‘r’ = vb‘i’[1,‘=‘r”] in ‘i’

}

}

gen lci2=eb2-1.96*sqrt(se2n*se2n+vb2)

gen uci2=eb2 + 1.96*sqrt(se2n*se2n+vb2)

gen pb2=exp(eb2)

gen plci2=exp(lci2)

gen puci2=exp(uci2)

*** meta-regression ***

metareg b2 b1, wsse(se2)

predict pb2r, xb

predict pse2r, stdp

gen epb2r=exp(pb2r)

gen plci2r=exp(pb2r-1.96*sqrt(pse2r*pse2r+se2n*se2n))

gen puci2r=exp(pb2r+1.96*sqrt(pse2r*pse2r+se2n*se2n))

}

### Appendix 1.7. R code for the simulation

sd1<-0.36

sd2<-0.15

eta1<- -0.5253

lambda0<- 0.0101

lambda1<- 0.4793

shift<-0

delta<-sd1*shift

sh2<-0.97

rt2<-0.03

sc2<-1/rt2

sh1<-1.13

rt1<-0.01

sc1<-1/rt1

rho.min<- -0.11

rho.max<- 0.186

var.pop.rel<-2.2

var.pop.dis<-17.2

m1<-m2<-rho<-n1<-n2<-s1<-s2<-var1<-var2<-prec1<-prec2<-matrix(,10)

sigma < - array(matrix(0,2,2),10)

y.val<-matrix(,10,2)

for (i in 1:10){

ll<-5

while (ll>0) {

m1[i]<-rnorm(1,eta1+delta,sd1)

m2[i]<-rnorm(1,lambda0+lambda1*m1[i],sd2)

rho[i]<-runif(1,rho.min,rho.max)

prec1[i]<-rgamma(1,shape=sh1,scale = sc1)

prec2[i]<-rgamma(1,shape=sh2,scale = sc2)

var1[i]<-1/prec1[i]

var2[i]<-1/prec2[i]

s1[i]<-sqrt(var1[i])

s2[i]<-sqrt(var2[i])

n1[i]<-round(runif(1,25,1000))

n2[i]<-n1[i]

sigma<- matrix(c(var1[i],s1[i]*s2[i]*rho[i],s1[i]*s2[i]*rho[i],var2[i]),2,2)

y.val[i,]<-mvrnorm(n=1, c(m1[i],m2[i]), sigma)

l1<-(s1[i]>3.0)

l2<-(s2[i]>3.0)

ll<-l1+l2

}

}

## Appendix 2 Predictions from sensitivity analysis using *t*-distribution

**Table A 2.1.** Predictions obtained from BRMA PTDF models (and BRMA PNF for comparison) for all studies in the ‘Sormani data’.

	Disability progression rate ratio, mean (95% CrI)				
	Paty (1)	Paty (2)	Miligan	Johnson	Jacobs/Simon
Observed	1.00 (0.67, 1.49)	0.71 (0.45, 1.12)	1.14 (0.26, 5.03)	0.88 (0.57, 1.35)	0.63 (0.38, 1.05)
BRMA PNF	0.98 (0.63, 1.53)	0.83 (0.51, 1.36)	0.86 (0.19, 3.96)	0.86 (0.54, 1.39)	0.85 (0.49, 1.47)
BRMA PTDF (4 df)	0.98 (0.62, 1.55)	0.83 (0.51, 1.37)	0.86 (0.19, 3.97)	0.86 (0.53, 1.39)	0.85 (0.49, 1.47)
BRMA PTDF (15 df)	0.98 (0.63, 1.53)	0.84 (0.51, 1.36)	0.88 (0.19, 4.05)	0.86 (0.53, 1.39)	0.85 (0.49, 1.46)
BRMA PTDF (30 df)	0.98 (0.63, 1.53)	0.84 (0.51, 1.36)	0.87 (0.19, 4.01)	0.86 (0.53, 1.39)	0.84 (0.49, 1.46)
	Fazekas	Millefiorini	Achiron	Li (1)	Li (2)

Observed	0.70 (0.36, 1.35)	0.19 (0.05, 0.79)	0.82 (0.19, 3.50)	0.81 (0.61, 1.08)	0.73 (0.54, 0.99)
BRMA PNF	0.66 (0.33, 1.33)	0.64 (0.15, 2.75)	0.65 (0.15, 2.86)	0.87 (0.62, 1.23)	0.85 (0.60, 1.21)
BRMA PTDF (4 df)	0.66 (0.32, 1.35)	0.64 (0.15, 2.77)	0.66 (0.15, 2.94)	0.87 (0.61, 1.23)	0.86 (0.60, 1.23)
BRMA PTDF (15 df)	0.66 (0.33, 1.33)	0.64 (0.15, 2.74)	0.66 (0.16, 2.89)	0.87 (0.62, 1.23)	0.85 (0.60, 1.21)
BRMA PTDF (30 df)	0.66 (0.33, 1.34)	0.64 (0.15, 2.75)	0.66 (0.15, 2.88)	0.87 (0.61, 1.22)	0.85 (0.60, 1.21)
	Clanet	Durelli	Baumhackl	Polman	Rudick

Observed	1.00 (0.83, 1.20)	0.43 (0.24, 0.78)	1.07 (0.74, 1.57)	0.59 (0.46, 0.75)	0.79 (0.65, 0.96)
BRMA PNF	1.09 (0.82, 1.45)	0.87 (0.47, 1.63)*	0.94 (0.61, 1.43)	0.57 (0.40, 0.82)	0.66 (0.50, 0.87)
BRMA PTDF (4 df)	1.08 (0.81, 1.45)	0.87 (0.46, 1.62)*	0.93 (0.60, 1.45)	0.57 (0.39, 0.83)	0.66 (0.50, 0.88)
BRMA PTDF (15 df)	1.08 (0.81, 1.44)	0.87 (0.46, 1.62)*	0.93 (0.61, 1.43)	0.57 (0.39, 0.82)	0.66 (0.50, 0.87)
BRMA PTDF (30 df)	1.08 (0.82, 1.43)	0.87 (0.46, 1.62)*	0.94 (0.61, 1.44)	0.57 (0.39, 0.82)	0.66 (0.50, 0.87)
	Coles (1)	Coles (2)	Mikol	Comi (1)	Comi (2)

Observed	0.35 (0.16, 0.74)	0.38 (0.19, 0.77)	1.34 (0.88, 2.06)	0.69 (0.52, 0.93)	0.73 (0.55, 0.97)
BRMA PNF	0.61 (0.27, 1.35)	0.53 (0.25, 1.12)	1.01 (0.62, 1.64)	0.66 (0.45, 0.96)	0.68 (0.47, 0.98)
BRMA PTDF (4 df)	0.60 (0.27, 1.36)	0.52 (0.24, 1.14)	1.01 (0.61, 1.64)	0.66 (0.45, 0.97)	0.68 (0.47, 0.99)
BRMA PTDF (15 df)	0.61 (0.27, 1.35)	0.53 (0.25, 1.14)	1.00 (0.62, 1.62)	0.66 (0.45, 0.96)	0.68 (0.47, 0.98)
BRMA PTDF (30 df)	0.60 (0.27, 1.35)	0.53 (0.25, 1.13)	1.01 (0.62, 1.63)	0.66 (0.45, 0.96)	0.68 (0.48, 0.98)
	Havrdova (1)	Havrdova (2)	Sorensen	O'Connor (1)	O'Connor (2)

Observed	1.23 (0.58, 2.62)	1.04 (0.48, 2.67)	0.64 (0.32, 1.28)	1.05 (0.84, 1.31)	1.10 (0.88, 1.37)
BRMA PNF	0.95 (0.43, 2.08)	0.85 (0.38, 1.90)	0.65 (0.31, 1.37)	1.06 (0.77, 1.45)	1.00 (0.74, 1.35)
BRMA PTDF (4 df)	0.94 (0.43, 2.06)	0.85 (0.38, 1.90)	0.66 (0.31, 1.40)	1.06 (0.76, 1.49)	0.99 (0.73, 1.35)
BRMA PTDF (15 df)	0.95 (0.43, 2.08)	0.85 (0.38, 1.90)	0.65 (0.31, 1.43)	1.06 (0.77, 1.46)	1.00 (0.73, 1.35)
BRMA PTDF (30 df)	0.94 (0.43, 2.06)	0.85 (0.38, 1.90)	0.65 (0.31, 1.44)	1.06 (0.77, 1.45)	0.99 (0.74, 1.34)

**Table A 2.2.** Predictions obtained from BRMA PTDF models (and BRMA PNF for comparison) for all studies in the ‘Oba data’.

Overall survival, mean (95% CrI)					
	**Historical trials**				
	FFCD-8801	NSAS-GC	JCOG-9206-1	JCOG-8801	SWOG-7804
Observed	0.84 (0.62, 1.14)	0.51 (0.29, 0.90)	0.60 (0.31, 1.18)	0.82 (0.54, 1.27)	0.93 (0.70, 1.24)
BRMA PNF	0.87 (0.60, 1.26)	0.86 (0.47, 1.57)	0.87 (0.43, 1.75)	0.87 (0.54, 1.39)	0.86 (0.60, 1.24)
BRMA PTDF (4 df)	0.87 (0.59, 1.27)	0.86 (0.46, 1.60)	0.87 (0.43, 1.77)	0.87 (0.53, 1.43)	0.86 (0.60, 1.25)
BRMA PTDF (15 df)	0.87 (0.60, 1.27)	0.86 (0.47, 1.58)	0.87 (0.43, 1.75)	0.86 (0.54, 1.40)	0.86 (0.60, 1.23)
BRMA PTDF (30 df)	0.87 (0.60, 1.27)	0.86 (0.46, 1.58)	0.87 (0.43, 1.76)	0.87 (0.54, 1.40)	0.86 (0.60, 1.23)
	EORTC-40813	Tsavaris	ICCG-1/81	ITMO	GITSG-8174

Observed	0.85 (0.64, 1.14)	0.55 (0.33, 0.89)	0.85 (0.64, 1.13)	0.98 (0.70, 1.37)	0.74 (0.53, 1.04)
BRMA PNF	0.87 (0.61, 1.25)	0.86 (0.50, 1.48)	0.87 (0.61, 1.24)	0.86 (0.58, 1.28)	0.87 (0.58, 1.29)
BRMA PTDF (4 df)	0.86 (0.50, 1.25)	0.87 (0.50, 1.50)	0.87 (0.60, 1.28)	0.86 (0.57, 1.29)	0.86 (0.58, 1.29)
BRMA PTDF (15 df)	0.86 (0.60, 1.24)	0.86 (0.50, 1.48)	0.87 (0.61, 1.24)	0.86 (0.58, 1.28)	0.87 (0.58, 1.29)
BRMA PTDF (30 df)	0.86 (0.59, 1.24)	0.86 (0.50, 1.47)	0.87 (0.61, 1.24)	0.86 (0.58, 1.28)	0.86 (0.58, 1.29)
	NCTTG-794151	ECCOG-EST3275	EORTC-40905	ICCG	

Observed	1.02 (0.69, 1.51)	0.94 (0.68, 1.30)	0.93 (0.64, 1.37)	1.05 (0.74, 1.49)	
BRMA PNF	0.86 (0.55, 1.34)	0.86 (0.58, 1.27)	0.86 (0.56, 1.33)	0.87 (0.58, 1.32)	
BRMA PTDF (4 df)	0.86 (0.55, 1.37)	0.86 (0.58, 1.29)	0.87 (0.55, 1.37)	0.87 (0.57, 1.33)	
BRMA PTDF (15 df)	0.86 (0.55, 1.35)	0.86 (0.58, 1.27)	0.86 (0.55, 1.34)	0.87 (0.58, 1.31)	
BRMA PTDF (30 df)	0.86 (0.55, 1.35)	0.86 (0.58, 1.27)	0.86 (0.56, 1.34)	0.87 (0.58, 1.31)	

	**Validation trials**				
	A-cirera	B-CLASSIC	E-GOIM-9602	F-GOIRC	

Observed	0.60 (0.39, 0.93)	0.72 (0.52, 1.00)	0.91 (0.69, 1.21)	0.90 (0.64, 1.26)	
BRMA PNF	0.85 (0.52, 1.39)	0.77 (0.50, 1.19)	0.88 (0.62, 1.24)	0.87 (0.59, 1.29)	
BRMA PTDF (4 df)	0.84 (0.51, 1.38)	0.74 (0.45, 1.21)	0.87 (0.61, 1.24)	0.87 (0.58, 1.30)	
BRMA PTDF (15 df)	0.84 (0.51, 1.37)	0.76 (0.49, 1.15)	0.87 (0.62, 1.22)	0.87 (0.58, 1.30)	
BRMA PTDF (30 df)	0.84 (0.52, 1.37)	0.76 (0.48, 1.20)	0.87 (0.63, 1.22)	0.87 (0.58, 1.29)	

## Appendix 3 Predictions from the frequentist models

**Table A 3.1.** Predictions obtained from the two frequentist models for all studies in the ‘Sormani data’.

	Disability progression rate ratio, mean (95% CrI)				
	Paty (1)	Paty (2)	Miligan	Johnson	Jacobs/Simon
Observed	1.00 (0.67, 1.49)	0.71 (0.45, 1.12)	1.14 (0.26, 5.03)	0.88 (0.57, 1.35)	0.63 (0.38, 1.05)
Meta-regression	0.99 (0.66, 1.48)	0.84 (0.53, 1.33)	0.93 (0.21, 4.11)	0.87 (0.56, 1.35)	0.85 (0.51, 1.42)
BRMA	0.99 (0.65, 1.50)	0.84 (0.52, 1.35)	0.87 (0.19, 4.05)	0.87 (0.55, 1.37)	0.85 (0.50, 1.45)
	Fazekas	Millefiorini	Achiron	Li (1)	Li (2)

Observed	0.70 (0.36, 1.35)	0.19 (0.05, 0.79)	0.82 (0.19, 3.50)	0.81 (0.61, 1.08)	0.73 (0.54, 0.99)
Meta-regression	0.66 (0.34, 1.29)	0.61 (0.14, 2.55)	0.63 (0.15, 2.69)	0.87 (0.65, 1.17)	0.86 (0.63, 1.17)
BRMA	0.67 (0.34, 1.33)	0.65 (0.15, 2.80)	0.66 (0.15, 2.91)	0.87 (0.64, 1.18)	0.86 (0.62, 1.18)
	Clanet	Durelli	Baumhackl	Polman	Rudick

Observed	1.00 (0.83, 1.20)	0.43 (0.24, 0.78)	1.07 (0.74, 1.57)	0.59 (0.46, 0.75)	0.79 (0.65, 0.96)
Meta-regression	1.08 (0.87, 1.34)	0.88 (0.48, 1.59)*	0.94 (0.64, 1.39)	0.58 (0.43, 0.78)	0.66 (0.55, 0.83)
BRMA	1.07 (0.88, 1.29)	0.87 (0.47, 1.62)*	0.94 (0.63, 1.41)	0.62 (0.48, 0.81)	0.66 (0.54, 0.82)
	Coles (1)	Coles (2)	Mikol	Comi (1)	Comi (2)

Observed	0.35 (0.16, 0.74)	0.38 (0.19, 0.77)	1.34 (0.88, 2.06)	0.69 (0.52, 0.93)	0.73 (0.55, 0.97)
Meta-regression	0.58 (0.27, 1.26)	0.49 (0.24, 1.01)	1.03 (0.66, 1.60)	0.66 (0.48, 0.91)	0.69 (0.51, 0.93)
BRMA	0.62 (0.28, 1.37)	0.55 (0.27, 1.15)	1.01 (0.63, 1.60)	0.77 (0.48, 0.93)	0.69 (0.50, 0.95)
	Havrdova (1)	Havrdova (2)	Sorensen	O'Connor (1)	O'Connor (2)

Observed	1.23 (0.58, 2.62)	1.04 (0.48, 2.67)	0.64 (0.32, 1.28)	1.05 (0.84, 1.31)	1.10 (0.88, 1.37)
Meta-regression	0.96 (0.45, 2.05)	0.86 (0.39, 1.88)	0.63 (0.31, 1.27)	1.06 (0.83, 1.37)	1.00 (0.78, 1.27)
BRMA	0.95 (0.44, 2.06)	0.86 (0.38, 1.90)	0.66 (0.31, 1.39)	1.06 (0.83, 1.35)	1.00 (0.78, 1.27)

**Table A 3.2.** Predictions obtained from the two frequentist models for all studies in the ‘Oba data’.

Overall survival, mean (95% CrI)					
	**Historical trials**				
	FFCD-8801	NSAS-GC	JCOG-9206-1	JCOG-8801	SWOG-7804
Observed	0.84 (0.62, 1.14)	0.51 (0.29, 0.90)	0.60 (0.31, 1.18)	0.82 (0.54, 1.27)	0.93 (0.70, 1.24)
Meta-regression	0.87 (0.63, 1.19)	0.50 (0.26, 0.97)	0.65 (0.33, 1.25)	0.82 (0.53, 1.26)	0.91 (0.67, 1.24)
BRMA	0.86 (0.62, 1.19)	0.86 (0.50, 1.47)	0.85 (0.45, 1.62)	0.85 (0.55, 1.32)	0.86 (0.63, 1.17)
	EORTC-40813	Tsavaris	ICCG-1/81	ITMO	GITSG-8174

Observed	0.85 (0.64, 1.14)	0.55 (0.33, 0.89)	0.85 (0.64, 1.13)	0.98 (0.70, 1.37)	0.74 (0.53, 1.04)
Meta-regression	0.78 (0.57, 1.06)	0.58 (0.33, 1.02)	0.91 (0.67, 1.24)	0.93 (0.66, 1.31)	0.76 (0.53, 1.09)
BRMA	0.84 (0.61, 1.14)	0.85 (0.52, 1.40)	0.87 (0.63, 1.19)	0.86 (0.61, 1.20)	0.85 (0.59, 1.21)
	NCTTG-794151	ECCOG-EST3275	EORTC-40905	ICCG	

Observed	1.02 (0.69, 1.51)	0.94 (0.68, 1.30)	0.93 (0.64, 1.37)	1.05 (0.74, 1.49)	
Meta-regression	0.99 (0.65, 1.49)	0.93 (0.66, 1.31)	0.92 (0.62, 1.36)	1.11 (0.75, 1.66)	
BRMA	0.86 (0.57, 1.29)	0.86 (0.61, 1.22)	0.86 (0.57, 1.28)	0.86 (0.60, 1.23)	

	**Validation trials**				
	A-cirera	B-CLASSIC	E-GOIM-9602	F-GOIRC	

Observed	0.60 (0.39, 0.93)	0.72 (0.52, 1.00)	0.91 (0.69, 1.21)	0.90 (0.64, 1.26)	
Meta-regression	0.57 (0.35, 0.93)	**0.58 (0.41, 0.82)**	0.92 (0.68, 1.24)	0.96 (0.67, 1.38)	
BRMA	0.82 (0.52, 1.27)	0.79 (0.60, 1.05)	0.86 (0.64, 1.17)	0.87 (0.60, 1.25)	
